# Modeling mortality risk effects of cigarettes and smokeless tobacco: results from the National Health Interview Survey Linked Mortality File Data

**DOI:** 10.1186/s12889-021-11801-w

**Published:** 2021-09-29

**Authors:** Esther Salazar, Chunfeng Ren, Brian L. Rostron, Ghideon Solomon

**Affiliations:** Center for Tobacco Products, U.S. Food and Drug Administration, 11785 Beltsville Dr., Calverton, MD 20705 USA

**Keywords:** Mortality risk, Cigarettes, Smokeless tobacco

## Abstract

**Background:**

Cigarettes and smokeless tobacco (SLT) products are among a wide range of tobacco products that are addictive and pose a significant health risk. In this study, we estimated smoking- and SLT use-related mortality hazard ratios (HRs) among U.S. adults by sex, age group, and cause of death, for nine mutually exclusive categories of smoking and/or SLT use.

**Methods:**

We used data from the public-use National Health Interview Survey Linked Mortality with mortality follow-up through 2015. We used Cox proportional hazard models to estimate mortality HRs, adjusted by race/ethnicity, education, poverty level, body mass index, and tobacco-use status.

**Results:**

With never users as reference group, HRs for smoking-related diseases for male exclusive current smokers aged 35–64 and 65+ were 2.18 (95% confidence interval [CI]: 1.79–2.65), and 2.45 (95% CI: 2.14–2.79), respectively. Similar significant HR estimates were found for females and for all-cause mortality (ACM) and other-cause mortality (OCM) outcomes. HRs for exclusive current SLT users were only significant for males aged 35–64 for ACM (HR: 2.04, 95% CI: 1.27–3.27) and OCM (HR: 2.80, 95% CI: 1.50–5.25). HRs for users who switched from cigarettes to SLT products were significant for males aged 65+ for smoking-related diseases (HR: 2.06, 95% CI: 1.47–2.88), SLT-related diseases (HR: 1.99, 95% CI: 1.36–2.89), and ACM (HR: 1.63, 95% CI: 1.21–2.19).

**Conclusions:**

Male exclusive current SLT users aged 35–64 had a significant HR for ACM and OCM outcomes, suggesting that deaths not attributed to SLT use could be contributing to the ACM elevated HR for exclusive current SLT users.

**Supplementary Information:**

The online version contains supplementary material available at 10.1186/s12889-021-11801-w.

## Background

Cigarettes and smokeless tobacco (SLT) products are among a wide range of tobacco products that are addictive and pose a significant health risk in current and former users [1, 2]. In the United States (U.S.), although cigarette smoking rates among adults have decreased (from 23.1% in 2000 to 13.9% in 2018) [3, 4], sales, advertising and promotion of SLT have increased [5] primarily among male adults. Since mortality risk estimates associated with cigarettes and SLT use can change over time, and previous estimates for SLT have been limited by relatively small sample size, updated mortality risk estimates associated with the use of cigarettes and SLT are warranted to assess the mortality impact in the U.S. population and to inform regulatory activities.

Although smoking-attributable mortality risks have been extensively studied over several decades [6–8], comparable estimates for SLT have been more limited. Henley et al. [9] published estimates of mortality hazard ratios (HRs) for SLT users among participants in the American Cancer Society’s Cancer Prevention Studies I and II with enrollment in 1959 and 1982, respectively. They found increased risk among SLT users for both all-cause mortality and specific causes including heart disease, stroke, and to some extent cancer. More recently, Timberlake et al. [10] conducted a survival analysis using data from participants in the Tobacco Use Supplement to the Current Population Survey from 1985 to 2011 contained in the National Longitudinal Mortality Study (NLMS). They also found higher mortality risk for coronary heart disease among SLT users. Similar studies have been conducted using NLMS and National Health Interview Survey (NHIS)-Linked Mortality Files (LMF) [11–13].

This study aims to estimate mortality rates and mortality risk effects among current and former users of cigarettes and SLT products in the U.S. by sex, age group and cause of death using the most updated NHIS data linked with death certificate records from the National Death Index (NDI). To date, the latest released NHIS-LMF provide mortality follow-up data from the date of survey participation through December 31, 2015 [14]. For years in which there are tobacco use behavior data associated with both cigarettes and SLT use (1987, 1991, 1992, 1994, 1998, 2000, 2005, 2010, 2012–2014), we estimated mortality risks for nine mutually exclusive tobacco use categories.

This study has advantages compared to similar studies conducted using the NHIS-LMF data. For instance, Fisher et al. [11] used the NHIS-LMF data with mortality follow-up through December 31, 2011 to estimate mortality risks for smokers and SLT users; however, they reported results for adults (sex-combined) aged 18+. Also, Rodu et al. [12] analyzed the NHIS-LMF 2015 data, and reported mortality risks for only one analysis group of males aged 40–79, assuming that tobacco use did not change over participants’ lifetimes. We extended these two studies using NHIS-LMF 2015 data, providing updated mortality risk estimates by sex and age group, and assuming a 10-year mortality follow-up to reduce misclassification of participants’ tobacco-use status during the survival time.

## Methods

### Study population

We used the public-use 2015 NHIS-LMF datasets that contain mortality follow-up data of U.S. adult civilian noninstitutionalized population aged 18+ in which participants were followed from the date of survey participation through December 31, 2015. We pooled data from 11 survey years (1987, 1991, 1992, 1994, 1998, 2000, 2005, 2010, 2012–2014) where smoking and SLT (chewing tobacco, snuff, dip, snus, or dissolvable tobacco) use were self-reported (Additional file 1: Table S1, shows all reported SLT products during the study period). To account for changes in the sample design across pooled NHIS data, we adjusted the analytic weights by dividing each sample weight by the number of pooled years [15].

### Measures

#### Tobacco use status

We used participants’ self-reported tobacco use status at the time of interview to define current, former, and never users of cigarettes and SLT as follows. Never cigarette smokers had never smoked 100 cigarettes in their lifetime. Current cigarette smokers had smoked 100 cigarettes in their lifetime, and at the time of interview had smoked every day or some days or had quit smoking within the past 2 years (under the assumption that recent quitters have similar health risk as current smokers) [16, 17]. Former cigarette smokers had smoked 100 cigarettes in their lifetime but had quit smoking more than 2 years prior to the survey. Never SLT users had never used SLT or used it less than 20 times in their lifetime. Current SLT users had used it at least once or at least 20 times and used it every day or some days at the time of interview. Former SLT users had used it at least once or at least 20 times and did not use it at all at the time of interview. We defined nine mutually exclusive tobacco user groups who did not use any other tobacco products including pipe, hookah, e-cigarettes, bidi, and cigars (see Additional file 1: Table S1): (1) current smokers and SLT users (dual current users); (2) current smokers and former SLT users; (3) current smokers and never SLT users (exclusive current smokers); (4) former smokers and current SLT users; (5) former smokers and former SLT users; (6) former smokers and never SLT users (exclusive former smokers); (7) never smokers and current SLT users (exclusive current SLT users); (8) never smokers and former SLT users (exclusive former SLT users); (9) never users of both cigarettes and SLT.

#### Cause of death

We used the underlying leading cause of death variable (UCOD_LEADING) [18] to derive five mortality outcomes by combining cause-specific death categories, as described in Additional file 1: Table S2: (1) all-cause mortality (ACM), (2) smoking-related diseases [17, 19], (3) SLT-related diseases, (4) lung diseases excluding lung cancer, and (5) other-cause mortality (OCM).

#### Demographic characteristics

We considered the following individual and socioeconomic characteristics: age, sex, race/ethnicity (Hispanic, non-Hispanic white, non-Hispanic black, non-Hispanic other), education (some high school and below, high school graduate or equivalent, some college and above), poverty level [20, 21] (below poverty threshold, at or above poverty threshold), and body mass index (BMI; unit: kg/m^2^; categories: underweight, BMI < 18.5; normal weight, 18.5 ≤ BMI < 25; overweight, 25 ≤ BMI < 30; obese, BMI ≥ 30).

### Analytic sample

The initial sample size included 266,561 NHIS participants aged 18+ at the time of interview, after excluding 81,983 participants with missing information on cigarette use, SLT use or ever users of other tobacco products (including pipe, hookah, e-cigarettes, bidi, and cigars). From this data, the mortality follow-up period ranged from 0 to 29 years (median = 10.4 years, mean = 12.1 years, standard deviation = 9.3 years). Since participants’ tobacco-use status was only provided at the time of interview and it might change during the follow-up period, for sensitivity analysis purposes we truncated (right-censored) the follow-up period to a maximum of 5, 10, 15, 20, and up to 29 years (maximum follow-up), under the assumption that tobacco-use status remained the same during the survival time. We only reported results from the 10-year follow-up data since results from the sensitivity analysis did not differ substantially. Previous analyses indicate that middle-aged and older adults are most likely to have long-term established patterns of tobacco use or never use, and current tobacco users are beginning to die from tobacco use-related diseases [12, 22]; thus, we restricted our analyses to participants aged 35+. Our final analytic sample included 220,891 female and male participants aged 35+ with complete information on cigarette and SLT use and mortality follow-up data, including 22,515 all-cause deaths during the 10-year follow-up period, with 5571 deaths among 51,373 current smokers and 486 deaths among 3324 current SLT users.

### Statistical analysis

We analyzed the data in 2019–2020. For each tobacco-use status and mortality outcome, we calculated death rates per 100,000 person-years as the ratio of “number of weighted reported deaths” to “weighted person-years survived” [23, 24], as described in the Additional file 1.

We used Cox proportional hazard models to estimate mortality HRs by tobacco-use status, age group and sex, assuming that tobacco-use status remains the same during follow-up, and accounting for the data’s complex survey design [25, 26]. Models were fitted independently by sex, age group, and cause of death, and adjusted by race/ethnicity, education, poverty level, BMI, and tobacco-use status. In all models, “never tobacco users” was used as reference group to estimate mortality HRs. Although we acknowledged that smoking and SLT use behavior characteristics, such as duration and intensity of use, are important predictors for risk mortality [8, 27], they were not included as covariates in our models because of the lack of data for SLT users during the study period. We reported HRs, with their corresponding 95% CIs, by tobacco-use status, sex and age groups (35–64 and 65+). We checked the proportional hazard assumption by comparing two Cox models, one specifying a covariate with time-independent effects (as described above) and one adding a time-dependent interaction term assuming that the effect of a covariate varies over time [28], while still accounting for the data’s complex survey design. To avoid multicollinearity when adding the interaction terms, we opted for multiplying the time-independent covariate by (log *t* – mean(log *t*)) where *t* denotes the follow-up time. The validity of the proportionality assumption was assessed by testing the hypothesis that all coefficients associated with the time-dependent term are zero, using the Rao-Scott likelihood-ratio test for complex survey [29, 30].

We also conducted a sensitivity analysis comparing results from five follow-up periods: 5, 10, 15, 20, and up to 29 years. We opted for reporting results from the 10-year follow-up data for the following two reasons: [1] as a reference, we chose the median follow-up period (10.4 years), under the assumption that, up to 10 years of survival time, tobacco-use status reported at the time of interview remained the same [2]; although the HR point estimates for exclusive SLT users slightly decreased as follow-up period increased, the HR estimates (and their corresponding 95% CIs) for other tobacco-use statuses were not substantially different across the follow-up periods (see Additional file 1: Table S8).

We used SAS 9.4 software [31] for data preparation and descriptive analysis, and R 3.6.1 [32] for complex survey data analysis using the *survey* package, version 3.36 [33, 34].

## Results

### Descriptive analysis

Table 1 shows weighted demographic and socioeconomic characteristics of participants aged 35+ at the time of interview, by tobacco-use status (Additional file 1: Table S3, shows the corresponding 95% CIs estimated using the modified Wilson method [35, 36]). The average age for exclusive current SLT users (53.68, 95% CI: 52.63–54.73) was higher than for exclusive current smokers (51.41, 95% CI: 51.24–51.58). Dual users (current smokers and SLT users) had the lowest average age (48.75, 95% CI: 47.51–49.98) while exclusive former smokers had the highest average age (60.19, 95% CI: 59.97–60.41). Exclusive current SLT users, along with exclusive current and former smokers, were more likely to be non-Hispanic white and have less education (less than high school or high school diploma) and higher poverty level (at or above 100% poverty threshold). Never tobacco users tend to have higher education (some college and higher) than dual and exclusive current smokers and SLT users. Exclusive current SLT users tend to be obese (35.49, 95% CI: 31.58–39.6) compared to exclusive current smokers (21.25, 95% CI: 20.66–21.85).

**Table 1:** Demographic and socioeconomic characteristics (weighted estimates)^a^ for NHIS participants^b^ aged 35+ at the time of interview

Variables	Current Smokers			Former Smokers			Never Smokers		Never Tobacco Users	All
	Current SLT Users	Former SLT Users	Never SLT Users	Current SLT Users	Former SLT Users	Never SLT Users	Current SLT Users	Former SLT Users		
**Mean age in years**	48.75	48.99	51.41	56.29	57.53	60.19	53.68	50.51	54.97	55.23
**Sex** %										
Male	79.11	75.25	37.56	92.08	79.68	38.23	75.81	77.10	32.53	36.25
female	20.89	24.75	62.44	7.92	20.32	61.77	24.19	22.90	67.47	63.75
**Race/ethnicity** %										
Hispanic	3.16	5.18	9.24	1.30	5.62	9.15	2.14	4.84	14.00	11.85
Non-Hispanic white	85.38	80.95	73.51	88.25	85.54	79.28	77.35	82.23	67.47	71.37
Non-Hispanic black	9.80	11.33	13.5	7.80	7.58	8.39	18.00	11.10	12.00	11.56
Non-Hispanic other	1.66	2.54	3.74	2.65	1.27	3.18	2.52	1.83	6.54	5.23
**Education** %										
Less than high school	31.85	23.05	22.31	33.82	23.66	16.71	36.76	14.18	16.17	17.70
High school diploma	35.88	40.96	38.64	32.42	32.75	30.33	29.93	23.06	26.83	29.80
Some college and higher	32.28	35.75	38.5	33.5	43.48	52.46	33.04	62.44	56.36	51.91
Missing	0.00	0.24	0.56	0.26	0.11	0.50	0.28	0.32	0.64	0.58
**Poverty level** %										
At or above 100% threshold	76.94	76.96	73.05	78.77	83.89	79.29	72.32	82.39	77.99	77.39
Below 100% threshold	13.03	16.11	14.08	12.13	6.39	7.01	14.63	7.38	8.62	9.39
Missing	10.02	6.93	12.87	9.1	9.73	13.7	13.05	10.23	13.39	13.22
**Body mass index** (kg/m^2^) %										
Underweight (< 18.5)	1.60	2.58	2.96	0.96	0.46	1.22	1.04	0.50	1.53	1.71
Normal weight (18.5–24.9)	30.58	31.06	40.94	21.39	21.56	33.1	21.61	19.66	34.84	35.19
Overweight (25.0–29.9)	38.56	41.31	32.14	45.76	42.77	35.57	39.64	43.31	34.14	34.35
Obese (30+)	27.27	24.00	21.25	30.68	33.26	26.85	35.49	35.63	25.94	25.49
Missing	1.99	1.05	2.72	1.21	1.95	3.27	2.22	0.89	3.55	3.27

^a^Additional file 1: Table S3, shows the 95% CIs for these weighted estimates

^b^Excluding participants with missing tobacco-use status, poly-users, and users of other tobacco products including pipe, hookah, e-cigarettes, bidi, and cigars. Survey years: 1987, 1991, 1992, 1994, 1998, 2000, 2005, 2010, and 2012–2014

### Cause-specific mortality risk effects

Table 2 shows death rates (per 100,000 person-years) with their corresponding 95% CIs, and unweighted number of deaths by tobacco-use status and leading cause of death for both females and males aged 35+. For ACM, along with all other mortality outcomes, the highest death rate is associated with former smokers who switch to SLT use (hereinafter referred to as “switchers”). Also, death rates for dual users are lower compared to both exclusive current smokers and exclusive current SLT users, for ACM, smoking-related diseases and OCM. This could be due to the fact that dual users are on average 3 and 5 years younger than exclusive current smokers and SLT users, respectively (Table 2). Tables S4 and S5 in Additional file 1 show the death rates and the corresponding 95% CIs, by tobacco-use status and leading cause of death for males and females aged 35+, respectively.

**Table 2:** Death rates (per 100,000 person-years), lower and upper limits (LL, UL) of the 95% CIs, and number of deaths by leading cause of death among adults aged 35+ using a maximum of 10-year follow-up^a^

	Current Smokers			Former Smokers			Never Smokers		Never Tobacco Users	All
	Current SLT users	Former SLT users	Never SLT users	Current SLT users	Former SLT users	Never SLT users	Current SLT users	Former SLT users		
**Number of Participants**	810	1976	48,587	728	1512	36,755	1786	1949	126,788	220,891
**All-cause mortality**										
Rate	1260.2	1486.7	1467.3	2362.1	1954.9	2046.4	1839.5	918.8	1174.3	1398.0
95% CI: LL	920.2	1235.8	1411.8	1773.2	1609.8	1971.0	1508.0	722.9	1145.1	1371.7
95% CI: UL	1600.1	1737.6	1522.8	2951.0	2300.0	2121.8	2171.0	1114.8	1203.6	1424.3
Deaths	81	228	5262	131	229	5199	274	154	10,957	22,515
**Smoking-related diseases** ^b^										
Rate	926.8	893.5	963.9	1712.3	1343.4	1249.1	984.5	523.9	669.6	841.5
95% CI: LL	646.5	709.4	921.3	1190.1	1075.7	1191.0	780.0	374.8	648.5	821.8
95% CI: UL	1207.1	1077.6	1006.5	2234.5	1611.0	1307.3	1189.0	673.1	690.6	861.3
Deaths	63	144	3644	94	169	3385	173	95	6630	14,397
**SLT-related diseases** ^c^										
Rate	840.2	723.1	806.7	1497.4	1173.7	1062.2	935.3	488.4	614.4	740.8
95% CI: LL	566.5	557.5	768.1	995.9	917.1	1009.0	734.0	341.1	594.4	722.4
95% CI: UL	1113.9	888.8	845.4	1998.8	1430.3	1115.4	1136.5	635.6	634.4	759.3
Deaths	56	118	3086	79	144	2885	164	87	6113	12,732
**Lung diseases excluding lung cancer** ^d^										
Rate	81.8^*^	160.1	147.4	187.5	153.7	171.1	44.8^*^	34.3^*^	52.6	94.8
95% CI: LL	19.3	82.4	131.2	91.3	80.8	151.4	13.1	6.9	46.8	88.7
95% CI: UL	144.2	237.9	163.7	283.7	226.6	190.8	76.5	61.8	58.3	100.9
Deaths	7	26	558	15	25	500	9	8	517	1665
**Other-cause mortality** ^e^										
Rate	310.3	549.3	465.7	560.2	548.7	722.7	789.1	378.5	479.0	520.7
95% CI: LL	137.8	395.2	434.4	348.9	359.2	680.8	566.3	253.4	460.0	505.5
95% CI: UL	482.8	703.4	496.9	771.5	738.3	764.6	1011.8	503.7	498.0	535.9
Deaths	18	84	1618	37	60	1814	101	59	4327	8118

^a^ Excluding participants with missing tobacco-use status, poly-users, and users of other tobacco products including pipe, hookah, e-cigarettes, bidi, and cigars. Survey years: 1987, 1991, 1992, 1994, 1998, 2000, 2005, 2010, and 2012–2014

^b^ Smoking-related diseases: diseases of heart, malignant neoplasms, chronic lower respiratory diseases, cerebrovascular diseases, diabetes mellitus, and influenza and pneumonia

^c^ SLT-related diseases: diseases of heart, malignant neoplasms, cerebrovascular diseases, diabetes mellitus

^d^ Lung diseases excluding lung cancer: chronic lower respiratory diseases, and influenza and pneumonia

^e^ Other-cause mortality: accidents, Alzheimer’s disease, nephritis, nephrotic syndrome and nephrosis, and all other causes

^*^ Estimates with relative standard error greater than 30%. Due to unreliable precision, these estimates should be interpreted with caution

**Fig. 1:**
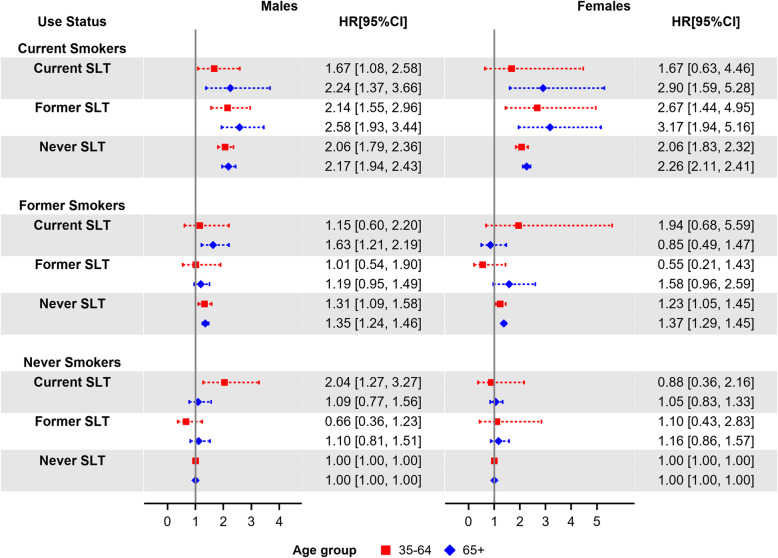
Estimated all-cause mortality hazard ratios by sex, age group (35–64 and 65+), and tobacco-use status (with never tobacco users as reference group), adjusted by race/ethnicity, education, poverty level, and BMI. Squares and diamonds indicate the point estimates for age groups 35–64 and 65+, respectively. Horizontal lines represent the length of the 95% CIs

For ACM, Fig. 1 shows the estimated HRs (with never users as reference group) and 95% CIs, by sex, age group (35–64 and 65+), and tobacco-use status. ACM HRs for male exclusive current smokers for age groups 35–64 and 65+ were 2.06 (95% CI: 1.79–2.36) and 2.17 (95% CI: 1.94–2.43), respectively; for male exclusive former smokers for the same age groups, the HRs were 1.31 (95% CI: 1.09–1.58) and 1.35 (95% CI: 1.24–1.46), respectively. ACM HRs for female exclusive current smokers for age groups 35–64 and 65+ were 2.06 (95% CI: 1.83–2.32) and 2.26 (95% CI: 2.11–2.41), respectively; for female exclusive former smokers for the same age groups, the HRs were 1.23 (95% CI: 1.05–1.45) and 1.37 (95% CI: 1.29–1.45), respectively. Higher values of ACM HRs for both males (HR: 2.58, 95% CI: 1.93–3.44) and females (HR: 3.17, 95% CI: 1.94–5.16) aged 65+ were associated with former SLT users who switched to cigarettes. The HR for dual users aged 35–64 was only significant for male users (HR: 1.67, 95% CI: 1.08–2.58), while the HR for dual users aged 65+ was significant for both, males (HR: 2.24, 95% CI: 1.37–3.66) and females (HR: 2.9, 95% CI: 1.59–5.28). ACM HRs for exclusive current SLT users were only significant for males aged 35–64 (HR: 2.04, 95% CI: 1.27–3.27). Additional file 1: Table S6, shows the estimated ACM HRs by tobacco-use status, sex, and 10-year age groups; ACM HRs for exclusive current SLT users were significant for male users aged 35–44 (HR: 2.64, 95% CI: 1.18–5.88), for adults (females and males combined) aged 35–44 (HR: 3.14, 95% CI: 1.44–6.87), and for adults aged 75–84 (HR: 1.34, 95% CI: 1–1.81). The ACM HR for switchers was only significant for males aged 65+ (HR: 1.63, 95% CI: 1.21–2.19).

**Fig. 2:**
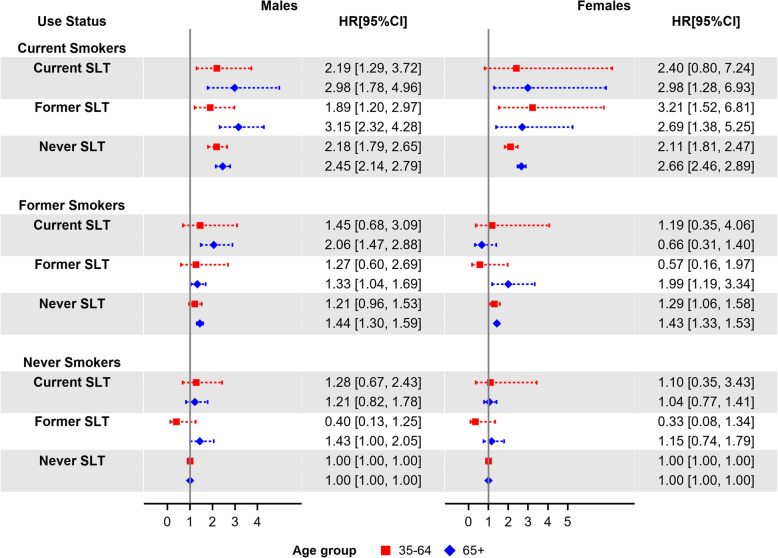
Estimated smoking-related mortality hazard ratios by sex, age group (35–64 and 65+), and tobacco-use status (with never tobacco users as reference group), adjusted by race/ethnicity, education, poverty level, and BMI. Squares and diamonds indicate the point estimates for age groups 35–64 and 65+, respectively. Horizontal lines represent the length of the 95% CIs

**Fig. 3:**
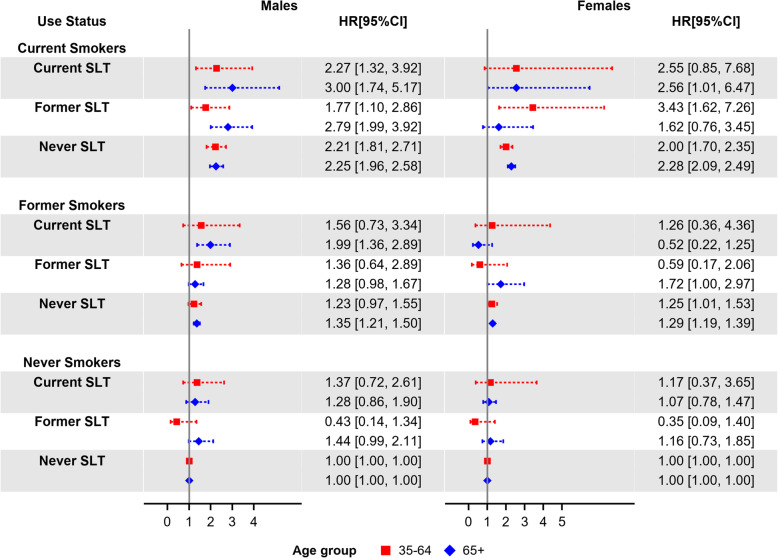
Estimated SLT-related mortality hazard ratios by sex, age group (35–64 and 65+), and tobacco-use status (with never tobacco users as reference group), adjusted by race/ethnicity, education, poverty level, and BMI. Squares and diamonds indicate the point estimates for age groups 35–64 and 65+, respectively. Horizontal lines represent the length of the 95% CIs

Figures 2 and 3 show the estimated mortality HRs for smoking-related and SLT-related diseases, respectively; HRs for current smokers, regardless of SLT use status, are comparable with the ACM HR estimates. HRs for exclusive current SLT users were not significant for these two mortality outcomes. Male exclusive former SLT users aged 65+ had borderline significant HRs of 1.43 (95% CI: 1.00–2.05) for smoking-related diseases and 1.44 (95% CI: 0.99–2.11) for SLT-related diseases. Additional file 1: Figure S1, shows estimated OCM HRs; male exclusive current SLT users aged 35–64 had a significant HR of 2.8 (95% CI: 1.50–5.25). OCM HRs for female and male exclusive current smokers were similar with the ACM HRs through all age groups. Additional file 1: Figure S2, shows the estimated HRs for lung diseases (excluding lung cancer); compared to other mortality outcomes, those estimates had wider 95% CIs due to the limited number of deaths from lung diseases.

Additional file 1: Table S7, shows the sex-combined HR estimates with their corresponding 95% CIs for all mortality outcomes for age groups 34–64 and 65+. For smoking-related diseases, the HR for switchers in the 65+ age category (HR: 2.28, 95% CI: 1.65–3.14) was significantly higher compared to exclusive former smokers (HR: 1.53, 95% CI: 1.44–1.62). For SLT-related diseases, and for the 65+ age group, we also observed a significant difference among switchers (HR: 2.16, 95% CI: 1.51–3.09) and exclusive former smokers (HR: 1.40, 95% CI: 1.32–1.49).

## Discussion

This analysis extends previous studies that used the NHIS-LMF data and provides updated mortality risk estimates by sex and tobacco-use status considering age groups (35–64, 65+, and 10-year groups), assuming a 10-year mortality follow-up to reduce misclassification of participants’ tobacco-use status during the survival time.

Our results are consistent with and contribute to previous findings on mortality risks attributable to cigarette and SLT use in the U.S. population. We observed increased mortality risk for cigarette smokers for all-cause and cause-specific mortality, consistent with previous research [7]. We also observed some evidence of increased mortality risk among exclusive SLT users, specifically for men aged 35–64 and from OCM. These results are generally consistent with previous estimates of increased ACM risk among daily SLT users [13] and sex-combined SLT users [12] that were obtained from NHIS-LMF data. Our results are also consistent with the previous observation of increased mortality risk for other causes among SLT users, especially in the 40–59 age group [12].

Our findings indicate that male exclusive current SLT users aged 35–64 had significant HRs for ACM and OCM. Also, the significant ACM HR was driven by a significant HR for male users aged 35–44. These results suggest that the elevated ACM HR among young male adults could be associated with other lifestyle factors (e.g., drug overdose, alcohol abuse) besides SLT use behaviors, disease-specific mortality associated with SLT use [10], or other unknown underlying factors that were not controlled for in this study. Male exclusive former SLT users aged 65+ had borderline significant HRs for both smoking-related and SLT-related diseases; however, sex-combined HR estimates were significant for these two mortality outcomes (see Additional file 1: Table S7). These results suggest that people may quit SLT use when they start experiencing major health conditions at older ages. Male dual users had elevated significant HRs for ACM, smoking-, and SLT-related diseases for age groups 35–64 and 65+; for these three mortality outcomes, HRs for female dual users were only significant in the 65+ age category. Sex-combined HRs estimates for dual users were significant for both age groups. For smoking- and SLT-related diseases, sex-combined mortality HRs were significantly higher for switchers aged 65+ compared to exclusive former smokers, with non-overlapping CIs between the two tobacco-use groups. These results were largely driven by the elevated HRs for male switchers and exclusive former smokers aged 65+. Although we observed significant risk differences between switchers and exclusive former smokers in older adults, those results did not account for smoking-related behavior for quitters (e.g., tobacco-use intensity, years of smoking, and quitting time) that may need to be considered to understand this finding.

Studies from other countries have shown that a large number of deaths worldwide were attributed to SLT use, with a large proportion in the Southeast Asian region [37–39]. Mortality estimates from nationally representative cohort studies, as presented in our study, would be beneficial in regions with high SLT-related mortality risk and prevalence.

### Limitations

This study is subject to the following limitations. First, self-reported tobacco-use data collected at the time of interview did not account for changes on tobacco-use status over participants’ lifetimes; thus, we used a maximum of 10-year mortality follow-up to reduce misclassification of participants’ tobacco-use status during the survival time. We also conducted a sensitivity analysis comparing results from various follow-up periods (5, 10, 15, 20, and up to 29 years), but results did not differ substantially (see Additional file 1: Table S8). Second, the sample size and number of reported deaths for exclusive current and former SLT users was limited due to low prevalence of SLT use across years; thus, we observed higher standard errors and wider 95% CIs compared to smokers. Third, this study did not include tobacco-use intensity, duration of use, quitting time, insurance status, and geographic information (urban/rural) as covariates in our models since these data were either not available for both cigarettes and SLT users in all the survey years analyzed or only available in the restricted-use NHIS data. Fourth, the criteria used to define former and never SLT users could potentially allow for misclassification of the two groups since it roughly categorized respondents who had ever used SLT less than 20 times in their lifetime and did not use it around the time of interview as former SLT users. Fifth, other health risk factors such as alcohol abuse, drug overdose, physical activities, hypertension, and diabetes were only available in some years of NHIS and were not controlled for in this study.

Further, although there were some changes in the SLT product landscape during the study period (such as the introduction of other forms of SLT like snus and dissolvables), this study did not explore how these product changes affect risk estimates. Lastly, although we presented mortality risk estimates by selected leading cause of death, our study did not explore disease-specific risk mortality outcomes (available in the restricted-use NHIS-LMF data). However, the low sample size for current and former SLT users would still be a limitation for disease-specific data analysis. Analysis of larger populations of SLT users and availability of data from more survey years, including SLT use behaviors, could enable more reliable conclusions on SLT-related mortality risk.

## Conclusions

For ACM, both male and female exclusive current smokers had significantly higher HRs than exclusive former smokers. For males aged 35–64, exclusive current SLT users had a significant ACM HR (2.04, 95% CI: 1.27–3.27), which was driven by an elevated HR for male exclusive current SLT users aged 35–44 (2.64, 95% CI: 1.18–5.88). Also, male exclusive current SLT users aged 35–64 had an elevated significant OCM HR (2.8, 95% CI: 1.5–5.25). Mortality HRs for female exclusive SLT users were not significant regardless of the underlying cause of death and likely due to the relatively small sample size for female SLT users. These results provide updated mortality risk estimates to assess the mortality impact of cigarettes and SLT products in the U.S. population, and may inform regulatory activities such as tobacco product standard development and product application reviews.

## Supplementary Information

**Additional file 1: Table S1.** Available tobacco products and data file type from NHIS data. **Table S2.** Underlying cause of death categories included on the public-use 2015 NHIS-LMF and derived mortality outcomes used for analysis. **Table S3.** Demographic and socioeconomic characteristics (weighted estimates and 95% CIs) for NHIS participants aged 35+ at the time of interview.^a^. **Table S4.** Death rates (per 100,000 person-years), 95% CIs, and number of deaths by leading cause of death among **male adults aged 35+** using a maximum of 10-year follow-up.^a^. **Table S5.** Death rates (per 100,000 person-years), 95% CIs, and number of deaths by leading cause of death among **female adults aged 35+** using a maximum of 10-year follow-up.^a^. **Table S6.** Estimated all-cause mortality hazard ratios (HRs) and 95% CIs by sex, 10-year age groups, and tobacco-use status (with never tobacco users as reference group), adjusted by race/ethnicity, education, poverty level, and BMI. Maximum mortality follow-up: 10 years. **Table S7.** Sex-combined hazard ratio (HR) estimates and 95% CIs by mortality outcome, age groups, and tobacco-use status (with never tobacco users as reference group), adjusted by race/ethnicity, education, poverty level, and BMI. Maximum mortality follow-up: 10 years. **Table S8.** Estimated all-cause mortality hazard ratios (HRs) and 95% CIs by years of follow-up, sex, age groups, and tobacco-use status (with never tobacco users as reference group), adjusted by race/ethnicity, education, poverty level, and BMI. **Figure S1.** Estimated other-cause mortality hazard ratios by sex, age group (35-64 and 65+), and tobacco-use status (with never tobacco users as reference group), adjusted by race/ethnicity, education, poverty level, and BMI. Squares and diamonds indicate the point estimates for age groups 35-64 and 65+, respectively. Horizontal lines represent the length of the 95% CIs. **Figure S2.** Estimated mortality hazard ratios for lung diseases (excluding lung cancer) by sex, age group (35-64 and 65+), and tobacco-use status (with never tobacco users as reference group), adjusted by race/ethnicity, education, poverty level, and BMI. Squares and diamonds indicate the point estimates for age groups 35-64 and 65+, respectively. Horizontal lines represent the length of the 95% CIs.

## Data Availability

The 2015 Public-Use Linked Mortality Files used and analyzed during the current study are publicly available through the Centers for Disease Control and Prevention website: https://www.cdc.gov/nchs/data-linkage/mortality-public.htm

## Abbreviations

SLT: Smokeless tobacco
HR: Hazard ratio
ACM: All-cause mortality
OCM: Other-cause mortality
NLMS: National Longitudinal Mortality Study
NDI: National Death Index
NHIS: National Health Interview Survey
LMF: Linked Mortality Files
BMI: Body mass index
CI: Confidence interval
