# Correction to: Modeling mortality risk effects of cigarettes and smokeless tobacco: results from the national health interview survey linked mortality file data

**DOI:** 10.1186/s12889-021-12242-1

**Published:** 2022-01-04

**Authors:** Esther Salazar, Chunfeng Ren, Brian L. Rostron, Ghideon Solomon

**Affiliations:** Center for Tobacco Products, U.S. Food and Drug Administration, 11785 Beltsville Dr, Calverton, MD 20705 USA

**Correction to: BMC Public Health 21, 1773 (2021)**

**https://doi.org/10.1186/s12889-021-11,801-w**

It was highlighted that the in the original article [1] in Table 1 and Table S3, the labels for the "Poverty level" variable (Below 100% threshold, At or above 100% threshold) were presented in the incorrect order. That is, label "At or above 100% threshold" should have appeared first. Also, in the Results section, the following sentence should have appeared as: "Exclusive current SLT users, along with exclusive current and former smokers, were more likely to be non-Hispanic white and have less education (less than high school or high school diploma) and **higher poverty level (at or above 100% poverty threshold)**." The original article has been updated.

**Table 1:** Demographic and socioeconomic characteristics (weighted estimates)^a^ for NHIS participants^b^ aged 35+ at the time of interview

Variables	Current Smokers			Former Smokers			Never Smokers		Never Tobacco Users	All
	Current SLT Users	Former SLT Users	Never SLT Users	Current SLT Users	Former SLT Users	Never SLT Users	Current SLT Users	Former SLT Users		
**Mean age in years**	48.75	48.99	51.41	56.29	57.53	60.19	53.68	50.51	54.97	55.23
**Sex** %										
Male	79.11	75.25	37.56	92.08	79.68	38.23	75.81	77.10	32.53	36.25
female	20.89	24.75	62.44	7.92	20.32	61.77	24.19	22.90	67.47	63.75
**Race/ethnicity** %										
Hispanic	3.16	5.18	9.24	1.30	5.62	9.15	2.14	4.84	14.00	11.85
Non-Hispanic white	85.38	80.95	73.51	88.25	85.54	79.28	77.35	82.23	67.47	71.37
Non-Hispanic black	9.80	11.33	13.5	7.80	7.58	8.39	18.00	11.10	12.00	11.56
Non-Hispanic other	1.66	2.54	3.74	2.65	1.27	3.18	2.52	1.83	6.54	5.23
**Education** %										
Less than high school	31.85	23.05	22.31	33.82	23.66	16.71	36.76	14.18	16.17	17.70
High school diploma	35.88	40.96	38.64	32.42	32.75	30.33	29.93	23.06	26.83	29.80
Some college and higher	32.28	35.75	38.5	33.5	43.48	52.46	33.04	62.44	56.36	51.91
Missing	0.00	0.24	0.56	0.26	0.11	0.50	0.28	0.32	0.64	0.58
**Poverty level** %										
At or above 100% threshold	76.94	76.96	73.05	78.77	83.89	79.29	72.32	82.39	77.99	77.39
Below 100% threshold	13.03	16.11	14.08	12.13	6.39	7.01	14.63	7.38	8.62	9.39
Missing	10.02	6.93	12.87	9.1	9.73	13.7	13.05	10.23	13.39	13.22
**Body mass index** (kg/m^2^) %										
Underweight (< 18.5)	1.60	2.58	2.96	0.96	0.46	1.22	1.04	0.50	1.53	1.71
Normal weight (18.5–24.9)	30.58	31.06	40.94	21.39	21.56	33.1	21.61	19.66	34.84	35.19
Overweight (25.0–29.9)	38.56	41.31	32.14	45.76	42.77	35.57	39.64	43.31	34.14	34.35
Obese (30+)	27.27	24.00	21.25	30.68	33.26	26.85	35.49	35.63	25.94	25.49
Missing	1.99	1.05	2.72	1.21	1.95	3.27	2.22	0.89	3.55	3.27

^a^ Additional file 1: Table S3, shows the 95% CIs for these weighted estimates

^b^ Excluding participants with missing tobacco-use status, poly-users, and users of other tobacco products including pipe, hookah, e-cigarettes, bidi, and cigars. Survey years: 1987, 1991, 1992, 1994, 1998, 2000, 2005, 2010, and 2012–2014

## Supplementary Information

**Additional file 1: Table S3**. Demographic and socioeconomic characteristics (weighted estimates and 95% CIs) for NHIS participants aged 35+ at the time of interview.^a^
