# Supplementary material for: Modeling mortality risk effects of cigarettes and smokeless tobacco: results from the National Health Interview Survey Linked Mortality File Data
Source: BMC Public Health. 2021 Sep 29;21:1773. doi: 10.1186/s12889-021-11801-w (PMC8482579; doi:10.1186/s12889-021-11801-w)
Supplement: Supplementary file 1 — Additional file 1: Table S1. Available tobacco products and data file type from NHIS data. Table S2. Underlying cause of death categories included on the public-use 2015 NHIS-LMF and derived mortality outcomes used for analysis. Table S3. Demographic and socioeconomic characteristics (weighted estimates and 95% CIs) for NHIS participants aged 35+ at the time of interview.a. Table S4. Death rates (per 100,000 person-years), 95% CIs, and number of deaths by leading cause of death among male adults aged 35+ using a maximum of 10-year follow-up.a. Table S5. Death rates (per 100,000 person-years), 95% CIs, and number of deaths by leading cause of death among female adults aged 35+ using a maximum of 10-year follow-up.a. Table S6. Estimated all-cause mortality hazard ratios (HRs) and 95% CIs by sex, 10-year age groups, and tobacco-use status (with never tobacco users as reference group), adjusted by race/ethnicity, education, poverty level, and BMI. Maximum mortality follow-up: 10 years. Table S7. Sex-combined hazard ratio (HR) estimates and 95% CIs by mortality outcome, age groups, and tobacco-use status (with never tobacco users as reference group), adjusted by race/ethnicity, education, poverty level, and BMI. Maximum mortality follow-up: 10 years. Table S8. Estimated all-cause mortality hazard ratios (HRs) and 95% CIs by years of follow-up, sex, age groups, and tobacco-use status (with never tobacco users as reference group), adjusted by race/ethnicity, education, poverty level, and BMI. Figure S1. Estimated other-cause mortality hazard ratios by sex, age group (35-64 and 65+), and tobacco-use status (with never tobacco users as reference group), adjusted by race/ethnicity, education, poverty level, and BMI. Squares and diamonds indicate the point estimates for age groups 35-64 and 65+, respectively. Horizontal lines represent the length of the 95% CIs. Figure S2. Estimated mortality hazard ratios for lung diseases (excluding lung cancer) by sex, age group (35-64 and [file 12889_2021_11801_MOESM1_ESM.docx]

**Modeling Mortality Risk Effects of Cigarettes and Smokeless Tobacco: Results from the National Health Interview Survey Linked Mortality File Data**

**(Supplementary Material)**

Esther Salazar, PhD; Chunfeng Ren, PhD; Brian L. Rostron, PhD; Ghideon Solomon, PhD

Center for Tobacco Products, U.S. Food and Drug Administration, MD, USA

**Estimation of death rates**

For each tobacco-use status and mortality outcome, we calculated death rates per 100,000 person-years as the ratio of “number of weighted reported deaths” to “weighted person-years survived” as follows. Let $y_{i}$ and $x_{i}$ be the death index (1=dead, 0=alive) and the survival time (number of years survived), respectively, for the $i^{\mathrm{th}}$ ($i=1,\cdots,m,$ and $m$ is the number of participants in the sample) sampled participant from the population in each tobacco-use status and mortality outcome. The estimator of death rate is $\hat{Y}/\hat{X}$, where $\hat{Y}=\sum_{i=1}^{m} w_{i}y_{i}$, $\hat{X}=\sum_{i=1}^{m} w_{i}x_{i}$, and $w_{i}$ is the $i^{\mathrm{th}}$ participant’s survey weight.^1,2^

**Table S1**: Available tobacco products and data file type from NHIS data.

| **Year** | **Data File Type** | **Tobacco Products** |
| --- | --- | --- |
| 1987 | Cancer control supplement  Cancer epidemiology | Cigarette, pipe, cigar, SLT (snuff, chewing tobacco) |
| 1991 | Health promotion and disease prevention | Cigarette, pipe, cigar, SLT (snuff, chewing tobacco) |
| 1992 | Cancer control supplement | Cigarette, pipe, cigar, SLT (snuff, chewing tobacco) |
| 1994 | Year 2000 objectives public use data | Cigarette, SLT (snuff, chewing tobacco) |
| 1998 | Sample adult person section  Sample adult prevention | Cigarette, pipe, cigar, SLT (snuff, chewing tobacco) |
| 2000 | Sample adult person section | Cigarette, pipe, cigar, SLT (snuff, chewing tobacco), bidis |
| 2005 | Cancer public use file  Sample adult person section | Cigarette, pipe, cigar, SLT (snuff, chewing tobacco), bidis |
| 2010 | Cancer public use file  Sample adult person section | Cigarette, cigar, SLT (snuff, chewing tobacco) |
| 2012-2013 | Sample adult public use file | Cigarette, SLT (chewing tobacco, snuff, dip, snus, or dissolvable tobacco^a^), other tobacco products (cigars, pipes, water pipes or hookahs, very small cigars that look like cigarettes, bidis or cigarillos) |
| 2014 | Sample adult public use file | Cigarette, SLT (chewing tobacco, snuff, dip, snus, or dissolvable tobacco^a^), e-cigarettes, and other tobacco products (cigars, pipes, water pipes or hookahs, very small cigars that look like cigarettes, bidis or cigarillos) |

^a^ Dissolvable tobacco products are finely ground tobacco pressed into shapes such as tablets, orbs, sticks, or strips and slowly dissolve in the mouth

NHIS: National Health Interview Survey

SLT: smokeless tobacco

**Table S2**: Underlying cause of death categories included on the public-use 2015 NHIS-LMF and derived mortality outcomes used for analysis.

| **Cause of Death (ICD-10 Codes) ^a^** | **Mortality Outcomes ^b^** | | | | |
| --- | --- | --- | --- | --- | --- |
|  | **All-Cause Mortality** | **Smoking-Related Diseases** | **SLT- Related**  **Diseases** | **Lung Diseases Excluding Lung Cancer** | **Other-Cause Mortality** |
| Diseases of heart (I00-I09, I11, I13, I20-I51) | X | X | X |  |  |
| Malignant neoplasms (C00-C97) | X | X | X |  |  |
| Chronic lower respiratory diseases (J40-J47) | X | X |  | X |  |
| Accidents (unintentional injuries) (V01-X59, Y85-Y86) | X |  |  |  | X |
| Cerebrovascular diseases (I60-I69) | X | X | X |  |  |
| Alzheimer’s disease (G30) | X |  |  |  | X |
| Diabetes mellitus (E10-E14) | X | X | X |  |  |
| Influenza and pneumonia (J10-J18) | X | X |  | X |  |
| Nephritis, nephrotic syndrome and nephrosis (N00-N07, N17-N19, N25-N27) | X |  |  |  | X |
| All other causes (residual) | X |  |  |  | X |

NHIS-LMF: National Health Interview Survey Linked Mortality Files

^a^ Source: Public-use NHIS-LMF 2015 data dictionary (<https://www.cdc.gov/nchs/data/datalinkage/public-use-2015-linked-mortality-files-data-dictionary.pdf>)

^b^ Each mortality outcome was derived by combining cause of death categories pointed by the symbol “X”.

SLT: smokeless tobacco

**Table S3**: Demographic and socioeconomic characteristics (weighted estimates and 95% CIs) for NHIS participants aged 35+ at the time of interview.^a^

| **Variables** | | **Current Smokers** | | | **Former Smokers** | | | **Never Smokers** | | **Never Tobacco Users** | **All** |
| --- | --- | --- | --- | --- | --- | --- | --- | --- | --- | --- | --- |
|  |  | **Current SLT Users** | **Former**  **SLT Users** | **Never SLT Users** | **Current SLT Users** | **Former SLT Users** | **Never**  **SLT Users** | **Current SLT Users** | **Former SLT Users** |  |  |
| **Mean age in years**  (95% CI) | | 48.75  [47.51-49.98] | 48.99  [48.21-49.76] | 51.41  [51.24-51.58] | 56.29  [54.87-57.72] | 57.53  [56.55-58.5] | 60.19  [59.97-60.41] | 53.68  [52.63-54.73] | 50.51  [49.77-51.25] | 54.97  [54.84-55.11] | 55.23  [55.12-55.34] |
| **Sex %** | |  |  |  |  |  |  |  |  |  |  |
|  | Male | 79.11  [73.78-83.60] | 75.25  [71.55-78.61] | 37.56  [36.82-38.30] | 92.08  [89.58-94.01] | 79.68  [76.28-82.71] | 38.23  [37.52-38.95] | 75.81  [72.34-78.98] | 77.1  [73.95-79.98] | 32.53  [32.11-32.95] | 36.25  [35.92-36.57] |
|  | Female | 20.89  [16.40-26.22] | 24.75  [21.39-28.45] | 62.44  [61.70-63.18] | 7.92  [5.99-10.42] | 20.32  [17.29-23.72] | 61.77  [61.05-62.48] | 24.19  [21.02-27.66] | 22.9  [20.02-26.05] | 67.47  [67.05-67.89] | 63.75  [63.43-64.08] |
| **Race/ethnicity %** | |  |  |  |  |  |  |  |  |  |  |
|  | Hispanic | 3.16  [1.91-5.17] | 5.18  [4.00-6.69] | 9.24  [8.81-9.70] | 1.30  [0.47-3.54] | 5.62  [4.11-7.64] | 9.15  [8.71-9.61] | 2.14  [1.28-3.56] | 4.84  [3.79-6.16] | 14.00  [13.58-14.42] | 11.85  [11.51-12.19] |
|  | Non-Hispanic white | 85.38  [81.73-88.4] | 80.95  [78.25-83.38] | 73.51  [72.83-74.18] | 88.25  [84.61-91.12] | 85.54  [82.98-87.77] | 79.28  [78.62-79.92] | 77.35  [73.79-80.55] | 82.23  [79.58-84.61] | 67.47  [66.93-68] | 71.37  [70.91-71.82] |
|  | Non-Hispanic black | 9.80  [7.41-12.85] | 11.33  [9.5-13.46] | 13.5  [12.98-14.04] | 7.80  [5.80-10.42] | 7.58  [6.09-9.4] | 8.39  [7.95-8.84] | 18  [15.29-21.06] | 11.1  [9.13-13.43] | 12.00  [11.63-12.37] | 11.56  [11.23-11.89] |
|  | Non-Hispanic other | 1.66  [0.74-3.68] | 2.54  [1.58-4.05] | 3.74  [3.43-4.08] | 2.65  [1.26-5.49] | 1.27  [0.68-2.33] | 3.18  [2.91-3.48] | 2.52  [1.44-4.35] | 1.83  [1.19-2.8] | 6.54  [6.26-6.83] | 5.23  [5.01-5.45] |
| **Education %** | |  |  |  |  |  |  |  |  |  |  |
|  | Less than high school | 31.85  [26.91-37.23] | 23.05  [20.58-25.71] | 22.31  [21.71-22.92] | 33.82  [29.15-38.82] | 23.66  [20.84-26.73] | 16.71  [16.16-17.27] | 36.76  [33.19-40.48] | 14.18  [12.19-16.44] | 16.17  [15.8-16.55] | 17.70  [17.39-18.03] |
|  | High school diploma | 35.88  [30.9-41.17] | 40.96  [37.34-44.67] | 38.64  [37.93-39.36] | 32.42  [27.64-37.59] | 32.75  [29.32-36.39] | 30.33  [29.69-30.98] | 29.93  [26.47-33.63] | 23.06  [19.94-26.50] | 26.83  [26.44-27.23] | 29.80  [29.47-30.14] |
|  | Some college and higher | 32.28  [26.93-38.14] | 35.75  [32.34-39.32] | 38.50  [37.77-39.23] | 33.50  [28.71-38.66] | 43.48  [39.76-47.27] | 52.46  [51.7-53.22] | 33.04  [29.29-37.02] | 62.44  [58.82-65.92] | 56.36  [55.84-56.88] | 51.91  [51.48-52.35] |
|  | Missing | 0  - | 0.24  [0.08-0.67] | 0.56  [0.46-0.68] | 0.26  [0.05-1.47] | 0.11  [0.02-0.47] | 0.50  [0.40-0.63] | 0.28  [0.10-0.74] | 0.32  [0.11-0.96] | 0.64  [0.57-0.71] | 0.58  [0.53-0.64] |
| **Poverty level %** | |  |  |  |  |  |  |  |  |  |  |
|  | At or above 100%  threshold | 76.94  [71.93-81.29] | 76.96  [74.01-79.67] | 73.05  [72.4-73.69] | 78.77  [74.34-82.62] | 83.89  [80.82-86.55] | 79.29  [78.68-79.89] | 72.32  [68.91-75.49] | 82.39  [79.91-84.61] | 77.99  [77.58-78.39] | 77.39  [77.05-77.73] |
|  | Below 100%  threshold | 13.03  [9.84-17.07] | 16.11  [13.69-18.86] | 14.08  [13.6-14.58] | 12.13  [9.14-15.92] | 6.39  [4.95-8.21] | 7.01  [6.68-7.37] | 14.63  [12.46-17.1] | 7.38  [5.95-9.13] | 8.62  [8.38-8.87] | 9.39  [9.18-9.61] |
|  | Missing | 10.02  [6.95-14.25] | 6.93  [5.46-8.75] | 12.87  [12.33-13.42] | 9.10  [6.66-12.31] | 9.73  [7.56-12.42] | 13.7  [13.18-14.22] | 13.05  [10.59-15.97] | 10.23  [8.40-12.41] | 13.39  [13.06-13.73] | 13.22  [12.94-13.51] |
| **Body mass index (kg/m^2^) %** | | |  |  |  |  |  |  |  |  |  |
|  | Underweight  (<18.5) | 1.60  [0.66-3.81] | 2.58  [1.70-3.91] | 2.96  [2.74-3.19] | 0.96  [0.34-2.68] | 0.46  [0.21-0.98] | 1.22  [1.09-1.36] | 1.04  [0.61-1.74] | 0.50  [0.25-1.00] | 1.53  [1.43-1.63] | 1.71  [1.64-1.79] |
|  | Normal weight  (18.5-24.9) | 30.58  [25.88-35.72] | 31.06  [27.82-34.49] | 40.94  [40.24-41.64] | 21.39  [17.52-25.84] | 21.56  [18.76-24.64] | 33.1  [32.39-33.81] | 21.61  [18.75-24.76] | 19.66  [17.23-22.34] | 34.84  [34.44-35.24] | 35.19  [34.87-35.5] |
|  | Overweight  (25.0-29.9) | 38.56  [33.47-43.92] | 41.31  [37.83-44.88] | 32.14  [31.47-32.82] | 45.76  [40.59-51.02] | 42.77  [38.86-46.78] | 35.57  [34.87-36.27] | 39.64  [35.9-43.51] | 43.31  [39.87-46.82] | 34.14  [33.75-34.54] | 34.35  [34.04-34.66] |
|  | Obese (30+) | 27.27  [22.55-32.57] | 24  [20.5-27.88] | 21.25  [20.66-21.85] | 30.68  [25.84-35.98] | 33.26  [29.5-37.25] | 26.85  [26.19-27.53] | 35.49  [31.58-39.6] | 35.63  [32.11-39.32] | 25.94  [25.57-26.31] | 25.49  [25.19-25.79] |
|  | Missing | 1.99  [0.89-4.39] | 1.05  [0.51-2.12] | 2.72  [2.49-2.96] | 1.21  [0.56-2.60] | 1.95  [0.98-3.85] | 3.27  [3.00-3.55] | 2.22  [1.51-3.26] | 0.89  [0.45-1.77] | 3.55  [3.39-3.71] | 3.27  [3.14-3.40] |
| ^a^ Excluding participants with missing tobacco-use status, poly-users, and users of other tobacco products including pipe, hookah, e-cigarettes, bidi, and cigars. Survey years: 1987, 1991, 1992, 1994, 1998, 2000, 2005, 2010, and 2012-2014  SLT: smokeless tobacco | | | | | | | | | | | |

| **Table S4**: Death rates (per 100,000 person-years), 95% CIs, and number of deaths by leading cause of death among **male adults aged 35+** using a maximum of 10-year follow-up.^a^ | | | | | | | | | | |
| --- | --- | --- | --- | --- | --- | --- | --- | --- | --- | --- |
|  | **Current Smokers** | | | **Former Smokers** | | | **Never Smokers** | | **Never Tobacco**  **Users** | **All** |
|  | **Current SLT**  **users** | **Former SLT**  **users** | **Never SLT**  **users** | **Current SLT**  **users** | **Former SLT**  **users** | **Never SLT**  **users** | **Current SLT**  **users** | **Former SLT**  **users** |  |  |
| **Number of participants** | 694 | 1,540 | 16,997 | 655 | 1,223 | 13,492 | 1,259 | 1,424 | 37,637 | 74,921 |
| **All-cause mortality** | |  |  |  |  |  |  |  |  |  |
| Rate  95% CI  Deaths | 1,089.08  [762.25-1,415.91]  62 | 1,403.75  [1,132.65-1,674.85]  178 | 1,585.20  [1,489.33-1,681.08]  2,017 | 2,312.71  [1,667.98-2,957.44]  111 | 2,041.25  [1,644.83-2,437.67]  189 | 2,465.62  [2,328.75-2,602.49]  2,189 | 1,242.10  [898.04-1,586.17]  105 | 537.12  [370.69-703.56]  70 | 974.95  [923.12-1,026.78]  2,523 | 1,393.80  [1,350.38-1,437.22]  7,444 |
| **Smoking-related diseases** ^b^ | |  |  |  |  |  |  |  |  |  |
| Rate  95% CI  Deaths | 815.36  [536.04-1,094.68]  47 | 849.09  [655.80-1,042.38]  116 | 997.35  [926.32-1,068.38]  1,354 | 1,763.73  [1,187.56-2,339.90]  84 | 1,380.32  [1,078.75-1,681.89]  139 | 1,529.49  [1,427.98-1,631.00]  1,468 | 622.78  [421.23-824.32]  66 | 344.67  [218.79-470.55]  48 | 556.00  [517.61-594.39]  1,501 | 846.72  [813.93-879.50]  4,823 |
| **SLT-related diseases** ^c^ | |  |  |  |  |  |  |  |  |  |
| Rate  95% CI  Deaths | 756.44  [481.54-1,031.34]  42 | 705.52  [524.49-886.55]  96 | 880.84  [814.37-947.31]  1,196 | 1,558.06  [1,004.45-2,111.67]  71 | 1,227.36  [932.15-1,522.57]  118 | 1,333.96  [1,240.73-1,427.19]  1,265 | 604.11  [404.48-803.74]  64 | 323.35  [198.67-448.03]  43 | 515.19  [478.82-551.55]  1,391 | 759.21  [728.71-789.71]  4,286 |
| **Lung diseases excluding lung cancer** ^d^ | | |  |  |  |  |  |  |  |  |
| Rate  95% CI  Deaths | 55.81*  [9.87-101.76]  5 | 135.53  [64.17-206.88]  20 | 108.75  [85.49-132.02]  158 | 178.44  [78.36-278.52]  13 | 138.01  [77.14-198.88]  21 | 175.69  [144.11-207.26]  203 | 17.47*  [0-43.21]^†^  2 | 20.81*  [2.08-39.54]  5 | 39.30  [29.83-48.77]  110 | 82.45  [73.29-91.62]  537 |
| **Other-cause mortality** ^e^ | | | |  | | |  | | | |
| Rate  95% CI  Deaths | 256.51  [105.77-407.26]  15 | 514.14  [340.84-687.44]  62 | 541.34  [487.27-595.42]  663 | 470.36  [258.03-682.68]  27 | 592.94  [371.49-814.39]  50 | 830.41  [752.92-907.90]  721 | 590.72  [320.68-860.76]  39 | 186.29  [88.16-284.43]  22 | 401.21  [368.52-433.90]  1,022 | 511.41  [485.62-537.19]  2,621 |
| ^a^ Excluding participants with missing tobacco status, poly-users and using other tobacco products including pipe, hookah, e-cigarettes, bidi, and cigars. Survey years: 1987, 1991, 1992, 1994, 1998, 2000, 2005, 2010, and 2012-2014  ^b^ Smoking-related diseases: diseases of heart, malignant neoplasms, chronic lower respiratory diseases, cerebrovascular diseases, diabetes mellitus, and influenza and pneumonia  ^c^ SLT-related diseases: diseases of heart, malignant neoplasms, cerebrovascular diseases, diabetes mellitus  ^d^ Lung diseases excluding lung cancer: chronic lower respiratory diseases, and influenza and pneumonia  ^e^ Other-cause mortality: accidents, Alzheimer’s disease, nephritis, nephrotic syndrome and nephrosis, and all other causes  * Estimates with relative standard error greater than 30%. Due to unreliable precision, these estimates should be interpreted with caution.  ^†^ Negative lower confidence limits were truncated at zero | | | | | | | | | | |

| **Table S5**: Death rates (per 100,000 person-years), 95% CIs, and number of deaths by leading cause of death among **female adults aged 35+** using a maximum of 10-year follow-up.^a^ | | | | | | | | | | |
| --- | --- | --- | --- | --- | --- | --- | --- | --- | --- | --- |
|  | **Current Smokers** | | | **Former Smokers** | | | **Never Smokers** | | **Never Tobacco**  **Users** | **All** |
|  | **Current SLT**  **users** | **Former SLT**  **users** | **Never SLT**  **users** | **Current SLT**  **users** | **Former SLT**  **users** | **Never SLT**  **users** | **Current SLT**  **users** | **Former SLT**  **users** |  |  |
| **Number of participants** | 116 | 436 | 31,590 | 73 | 289 | 23,263 | 527 | 525 | 89,151 | 145,970 |
| **All-cause mortality** | | | |  | | |  | | | |
| Rate  95% CI  Deaths | 2,403.81  [994.07-3,813.56]  19 | 1,807.95  [1,187.10-2,428.80]  50 | 1,402.07  [1,337.85-1,466.30]  3,245 | 2,851.53  [1,246.84-4,456.22]  20 | 1,624.95  [932.68-2,317.22]  40 | 1,805.93  [1,715.55-1,896.31]  3,010 | 3,810.75  [2,973.83-4,647.67]  169 | 2,261.32  [1,610.00-2,912.63]  84 | 1,269.48  [1,234.26-1,304.70]  8,434 | 1,400.29  [1,368.42-1,432.16]  15,071 |
| **Smoking-related diseases** ^b^ | |  |  |  |  |  |  |  |  |  |
| Rate  95% CI  Deaths | 1,669.41*  [576.19-2,762.64]  16 | 1,065.98  [572.87-1,559.10]  28 | 945.30  [893.67-996.92]  2,290 | 1,222.21*  [391.85-2,052.57]  10 | 1,201.63  [652.09-1,751.16]  30 | 1,087.07  [1,018.43-1,155.71]  1,917 | 2,129.37  [1,527.48-2,731.26]  107 | 1,141.35  [645.07-1,637.62]  47 | 723.56  [697.84-749.27]  5,129 | 838.66  [815.30-862.03]  9,574 |
| **SLT-related diseases** ^c^ | |  |  |  |  |  |  |  |  |  |
| Rate  95% CI  Deaths | 1,397.50*  [386.84-2,408.17]  14 | 790.96  [385.63-1,196.29]  22 | 765.60  [720.16-811.05]  1,890 | 922.53*  [235.98-1,609.07]  8 | 968.34  [499.51-1,437.16]  26 | 905.14  [844.25-966.02]  1,620 | 1,977.00  [1,386.49-2,567.52]  100 | 1,056.19  [571.94-1,540.43]  44 | 661.49  [637.21-685.78]  4,722 | 730.75  [709.04-752.46]  8,446 |
| **Lung diseases excluding lung cancer** ^d^ | | |  |  |  |  |  |  |  |  |
| Rate  95% CI  Deaths | 244.90*  [0-597.31]^†^  2 | 255.45*  [0-517.56]^†^  6 | 169.04  [147.18-190.90]  400 | 277.61*  [0-674.97]^†^  2 | 214.29*  [0-480.51]^†^  4 | 168.41  [143.06-193.75]  297 | 125.81*  [27.39-224.24]  7 | 79.38*  [0-180.68]^†^  3 | 58.84  [51.56-66.12]  407 | 101.64  [93.99-109.28]  1,128 |
| **Other-cause mortality** ^e^ | | | |  | | |  | | | |
| Rate  95% CI  Deaths | 648.15*  [0-1,472.98]^†^  3 | 684.05  [339.37-1,028.73]  22 | 423.72  [387.05-460.40]  955 | 1,481.30*  [342.61-2,619.98]  10 | 379.10*  [34.13-724.07]  10 | 659.89  [608.41-711.36]  1,093 | 1,395.82  [1,012.78-1,778.86]  62 | 1,030.29  [602.52-1,458.07]  37 | 515.82  [492.64-539.01]  3,305 | 525.79  [506.84-544.74]  5,497 |
| ^a^ Excluding participants with missing tobacco status, poly-users and using other tobacco products including pipe, hookah, e-cigarettes, bidi, and cigars. Survey years: 1987, 1991, 1992, 1994, 1998, 2000, 2005, 2010, and 2012-2014  ^b^ Smoking-related diseases: diseases of heart, malignant neoplasms, chronic lower respiratory diseases, cerebrovascular diseases, diabetes mellitus, and influenza and pneumonia  ^c^ SLT-related diseases: diseases of heart, malignant neoplasms, cerebrovascular diseases, diabetes mellitus  ^d^ Lung diseases excluding lung cancer: chronic lower respiratory diseases, and influenza and pneumonia  ^e^ Other-cause mortality: accidents, Alzheimer’s disease, nephritis, nephrotic syndrome and nephrosis, and all other causes  * Estimates with relative standard error greater than 30%. Due to unreliable precision, these estimates should be interpreted with caution  ^†^ Negative lower confidence limits were truncated at zero | | | | | | | | | | |
|  | | | | | | | | | | |

**Table S6**: Estimated all-cause mortality hazard ratios (HRs) and 95% CIs by sex, 10-year age groups, and tobacco-use status (with never tobacco users as reference group), adjusted by race/ethnicity, education, poverty level, and BMI. Maximum mortality follow-up: 10 years.

| **Sex** | **Age Group** | **Current Smokers** | | | | | | **Former Smokers** | | | | | | **Never Smokers** | | | |
| --- | --- | --- | --- | --- | --- | --- | --- | --- | --- | --- | --- | --- | --- | --- | --- | --- | --- |
|  |  | **Current SLT Users** | | **Former SLT Users** | | **Never SLT Users** | | **Current SLT Users** | | **Former SLT Users** | | **Never SLT Users** | | **Current SLT Users** | | **Former SLT Users** | |
|  |  | *(Dual Users)* | |  |  | *(Exclusive Current Smokers)* | | *(Switchers: cigarettes to SLT)* | |  |  | *(Exclusive Former Smokers)* | | *(Exclusive Current SLT Users)* | | *(Exclusive Former SLT Users)* | |
|  |  | **HR** | **95% CI** | **HR** | **95% CI** | **HR** | **95% CI** | **HR** | **95% CI** | **HR** | **95% CI** | **HR** | **95% CI** | **HR** | **95% CI** | **HR** | **95% CI** |
| **Males** |  |  |  |  |  |  |  |  |  |  |  |  |  |  |  |  |  |
|  | 35-44 | 1.28 | [0.46-3.56] | **1.97** | [1.01-3.81] | **1.62** | [1.19-2.2] | 0.46 | [0.06-3.32] | 2.19 | [0.73-6.59] | 1.00 | [0.66-1.52] | **2.64** | [1.18-5.88] | 0.99 | [0.41-2.36] |
|  | 45-54 | 1.34 | [0.68-2.63] | **2.41** | [1.45-4] | **1.88** | [1.49-2.37] | 1.27 | [0.36-4.49] | 0.51 | [0.17-1.49] | 1.34 | [0.94-1.92] | 1.58 | [0.73-3.38] | 0.49 | [0.14-1.66] |
|  | 55-64 | **2.70** | [1.43-5.11] | **1.96** | [1.16-3.3] | **2.55** | [2.09-3.11] | 1.34 | [0.64-2.81] | 0.90 | [0.36-2.3] | **1.46** | [1.14-1.87] | 1.76 | [0.81-3.79] | 0.48 | [0.13-1.75] |
|  | 65-74 | **2.53** | [1.34-4.78] | **3.32** | [2.39-4.61] | **2.45** | [2.05-2.92] | **1.96** | [1.11-3.46] | **1.62** | [1.04-2.53] | **1.39** | [1.17-1.65] | 1.56 | [0.79-3.09] | 0.90 | [0.47-1.72] |
|  | 75-84 | **2.29** | [1.07-4.9] | **2.53** | [1.77-3.63] | **2.34** | [1.97-2.78] | **1.85** | [1.16-2.95] | 1.34 | [0.95-1.87] | **1.47** | [1.29-1.67] | 1.40 | [0.83-2.36] | 1.38 | [0.81-2.34] |
|  | 85+ | **1.59** | [1.12-2.25] | 0.86 | [0.3-2.43] | **1.50** | [1.06-2.14] | 1.59 | [0.81-3.11] | 0.91 | [0.54-1.52] | **1.25** | [1.02-1.54] | 0.63 | [0.32-1.25] | 1.10 | [0.57-2.14] |
| **Females** |  |  |  |  |  |  |  |  |  |  |  |  |  |  |  |  |  |
|  | 35-44 | - | - | 2.93 | [0.76-11.3] | **1.40** | [1.05-1.86] | 7.28 | [0.8-65.83] | - | - | 0.61 | [0.38-0.98] | 0.28 | [0.04-2.06] | - | - |
|  | 45-54 | **3.95** | [1.13-13.76] | 1.25 | [0.41-3.81] | **2.17** | [1.75-2.68] | 5.20 | [0.92-29.31] | 0.24 | [0.03-1.74] | 1.13 | [0.82-1.56] | 1.72 | [0.27-11.13] | 0.63 | [0.15-2.63] |
|  | 55-64 | 0.96 | [0.25-3.75] | **3.60** | [1.55-8.35] | **2.29** | [1.95-2.68] | 0.68 | [0.13-3.6] | 0.90 | [0.31-2.62] | **1.43** | [1.17-1.75] | 0.79 | [0.34-1.85] | 1.96 | [0.62-6.14] |
|  | 65-74 | **4.61** | [1.99-10.66] | **4.31** | [2.24-8.3] | **2.68** | [2.38-3.02] | 1.51 | [0.54-4.21] | **2.27** | [1.01-5.08] | **1.60** | [1.4-1.83] | 1.01 | [0.56-1.83] | 0.95 | [0.4-2.29] |
|  | 75-84 | **2.48** | [1.15-5.35] | **2.40** | [1.08-5.3] | **2.38** | [2.15-2.64] | 0.35 | [0.11-1.14] | 1.37 | [0.64-2.96] | **1.41** | [1.29-1.54] | 1.16 | [0.81-1.66] | 1.39 | [0.81-2.38] |
|  | 85+ | 1.72 | [0.23-12.81] | 2.89 | [0.79-10.51] | **1.63** | [1.35-1.96] | 0.99 | [0.54-1.81] | 1.39 | [0.52-3.71] | **1.25** | [1.12-1.4] | 0.95 | [0.6-1.48] | 1.43 | [0.76-2.69] |
| **All** |  |  |  |  |  |  |  |  |  |  |  |  |  |  |  |  |  |
|  | 35-44 | 1.59 | [0.57-4.39] | **2.62** | [1.48-4.64] | **1.54** | [1.25-1.89] | 0.89 | [0.21-3.88] | 2.26 | [0.76-6.75] | 0.81 | [0.59-1.12] | **3.14** | [1.44-6.87] | 1.16 | [0.49-2.76] |
|  | 45-54 | **2.04** | [1.13-3.68] | **2.76** | [1.74-4.38] | **2.08** | [1.76-2.46] | 1.89 | [0.65-5.51] | 0.56 | [0.21-1.51] | 1.26 | [0.99-1.6] | 1.95 | [0.97-3.92] | 0.60 | [0.22-1.67] |
|  | 55-64 | **2.66** | [1.49-4.77] | **2.69** | [1.72-4.2] | **2.45** | [2.18-2.77] | 1.50 | [0.75-2.99] | 1.04 | [0.48-2.26] | **1.48** | [1.28-1.72] | 1.52 | [0.78-2.96] | 0.94 | [0.41-2.2] |
|  | 65-74 | **3.73** | [2.25-6.19] | **4.44** | [3.27-6.02] | **2.69** | [2.43-2.97] | **2.48** | [1.49-4.13] | **2.21** | [1.51-3.23] | **1.60** | [1.44-1.77] | 1.45 | [0.88-2.4] | 1.06 | [0.64-1.78] |
|  | 75-84 | **2.60** | [1.48-4.59] | **2.95** | [2.09-4.14] | **2.42** | [2.21-2.64] | **2.01** | [1.28-3.16] | **1.68** | [1.24-2.27] | **1.52** | [1.41-1.64] | **1.34** | [1-1.81] | **1.50** | [1.01-2.21] |
|  | 85+ | 1.88 | [0.81-4.37] | 1.29 | [0.52-3.2] | **1.61** | [1.36-1.89] | 1.62 | [0.99-2.67] | 1.21 | [0.78-1.88] | **1.32** | [1.2-1.45] | 0.86 | [0.59-1.24] | 1.35 | [0.83-2.2] |
| Boldface indicates statistical significance (p<0.05) | | | | | | | | | | | | | | | | | |

**Table S7**: Sex-combined hazard ratio (HR) estimates and 95% CIs by mortality outcome, age groups, and tobacco-use status (with never tobacco users as reference group), adjusted by race/ethnicity, education, poverty level, and BMI. Maximum mortality follow-up: 10 years.

| **Mortality Outcome** | **Age Group** | **Current Smokers** | | | | | | **Former Smokers** | | | | | | **Never Smokers** | | | |
| --- | --- | --- | --- | --- | --- | --- | --- | --- | --- | --- | --- | --- | --- | --- | --- | --- | --- |
|  |  | **Current SLT users** | | **Former SLT users** | | **Never SLT users** | | **Current SLT users** | | **Former SLT users** | | **Never SLT users** | | **Current SLT users** | | **Former SLT users** | |
|  |  | *(Dual users)* | |  |  | *(Exclusive current smokers)* | | *(Switchers: cigarettes to SLT)* | |  |  | *(Exclusive former smokers)* | | *(Exclusive current SLT users)* | | *(Exclusive former SLT users)* | |
|  |  | **HR** | **95% CI** | **HR** | **95% CI** | **HR** | **95% CI** | **HR** | **95% CI** | **HR** | **95% CI** | **HR** | **95% CI** | **HR** | **95% CI** | **HR** | **95% CI** |
| All-cause mortality | 35-64 | **2.12** | [1.43-3.15] | **2.73** | [2.06-3.63] | **2.11** | [1.93-2.31] | 1.52 | [0.85-2.69] | 1.10 | [0.62-1.95] | **1.30** | [1.16-1.46] | **2.16** | [1.4-3.34] | 0.89 | [0.53-1.49] |
|  | 65+ | **2.88** | [1.96-4.24] | **3.28** | [2.56-4.22] | **2.28** | [2.15-2.42] | **1.84** | [1.4-2.42] | **1.55** | [1.27-1.9] | **1.45** | [1.38-1.51] | 1.14 | [0.94-1.39] | 1.22 | [0.98-1.54] |
| Smoking-related diseases ^a^ | 35-64 | **2.67** | [1.66-4.3] | **2.52** | [1.71-3.7] | **2.19** | [1.94-2.47] | 1.76 | [0.87-3.54] | 1.32 | [0.67-2.62] | **1.28** | [1.11-1.48] | 1.42 | [0.81-2.48] | 0.44 | [0.17-1.15] |
|  | 65+ | **3.56** | [2.3-5.49] | **3.71** | [2.8-4.91] | **2.66** | [2.48-2.84] | **2.28** | [1.65-3.14] | **1.81** | [1.46-2.25] | **1.53** | [1.44-1.62] | 1.21 | [0.96-1.52] | **1.39** | [1.04-1.87] |
| SLT-related diseases ^b^ | 35-64 | **2.73** | [1.68-4.45] | **2.42** | [1.62-3.62] | **2.13** | [1.88-2.42] | 1.85 | [0.92-3.72] | 1.39 | [0.7-2.75] | **1.26** | [1.09-1.46] | 1.49 | [0.85-2.62] | 0.46 | [0.18-1.2] |
|  | 65+ | **3.39** | [2.13-5.4] | **3.04** | [2.24-4.13] | **2.33** | [2.16-2.51] | **2.16** | [1.51-3.09] | **1.70** | [1.35-2.15] | **1.40** | [1.32-1.49] | 1.26 | [0.99-1.61] | **1.40** | [1.03-1.92] |
| Lung diseases excluding lung cancer ^c^ | 35-64 | 1.66 | [0.22-12.52] | 4.21 | [1.05-16.88] | **3.21** | [2.05-5.01] | - | - | - | - | 1.69 | [0.87-3.26] | - | - | - | - |
|  | 65+ | **5.34** | [2.19-13.03] | **10.91** | [6.26-19.02] | **6.11** | [5.16-7.23] | **3.44** | [2.01-5.91] | **2.98** | [1.83-4.86] | **2.85** | [2.41-3.38] | 0.73 | [0.36-1.47] | 1.23 | [0.55-2.77] |
| Other-cause mortality ^d^ | 35-64 | 1.50 | [0.73-3.05] | **2.99** | [1.97-4.55] | **2.02** | [1.76-2.32] | 1.16 | [0.43-3.13] | 0.79 | [0.28-2.23] | **1.33** | [1.09-1.62] | **3.04** | [1.67-5.56] | 1.43 | [0.77-2.65] |
|  | 65+ | 1.66 | [0.72-3.8] | **2.53** | [1.72-3.73] | **1.66** | [1.49-1.84] | 1.13 | [0.73-1.75] | 1.15 | [0.78-1.71] | **1.32** | [1.22-1.43] | 1.05 | [0.77-1.43] | 0.98 | [0.66-1.46] |
| ^a^ Smoking-related diseases: diseases of heart, malignant neoplasms, chronic lower respiratory diseases, cerebrovascular diseases, diabetes mellitus, and influenza and pneumonia  ^b^ SLT-related diseases: diseases of heart, malignant neoplasms, cerebrovascular diseases, diabetes mellitus  ^c^ Lung diseases excluding lung cancer: chronic lower respiratory diseases, and influenza and pneumonia  ^d^ Other-cause mortality: accidents, Alzheimer’s disease, nephritis, nephrotic syndrome and nephrosis, and all other causes  Boldface indicates statistical significance (p<0.05) | | | | | | | | | | | | | | | | | |

**Table S8**: Estimated all-cause mortality hazard ratios (HRs) and 95% CIs by years of follow-up, sex, age groups, and tobacco-use status (with never tobacco users as reference group), adjusted by race/ethnicity, education, poverty level, and BMI.

| **Follow-up / Sex** | **Age Group** | **Current Smokers** | | | | | | **Former Smokers** | | | | | | **Never Smokers** | | | |
| --- | --- | --- | --- | --- | --- | --- | --- | --- | --- | --- | --- | --- | --- | --- | --- | --- | --- |
|  |  | **Current SLT users** | | **Former SLT users** | | **Never SLT users** | | **Current SLT users** | | **Former SLT users** | | **Never SLT users** | | **Current SLT users** | | **Former SLT users** | |
|  |  | *(Dual users)* | |  |  | *(Exclusive current smokers)* | | *(Switchers: cigarettes to SLT)* | |  |  | *(Exclusive former smokers)* | | *(Exclusive current SLT users)* | | *(Exclusive former SLT users)* | |
|  |  | **HR** | **95% CI** | **HR** | **95% CI** | **HR** | **95% CI** | **HR** | **95% CI** | **HR** | **95% CI** | **HR** | **95% CI** | **HR** | **95% CI** | **HR** | **95% CI** |
| **5-year follow-up** | |  |  |  |  |  |  |  |  |  |  |  |  |  |  |  |  |
| Males | 35-64 | 1.45 | [0.72-2.93] | **2.22** | [1.4-3.52] | **2.16** | [1.81-2.57] | 0.64 | [0.18-2.2] | 0.83 | [0.32-2.15] | **1.45** | [1.15-1.83] | **2.26** | [1.17-4.37] | 0.83 | [0.4-1.71] |
|  | 65+ | 1.60 | [0.91-2.82] | **2.57** | [1.85-3.57] | **2.21** | [1.91-2.57] | **1.75** | [1.17-2.64] | 1.30 | [0.96-1.75] | **1.34** | [1.2-1.5] | 0.96 | [0.63-1.45] | 0.99 | [0.64-1.52] |
| Females | 35-64 | 2.07 | [0.69-6.21] | **3.11** | [1.58-6.12] | **2.15** | [1.82-2.52] | 2.23 | [0.47-10.52] | 0.57 | [0.19-1.72] | **1.29** | [1.05-1.58] | 1.22 | [0.44-3.39] | 1.46 | [0.53-4] |
|  | 65+ | **3.35** | [1.51-7.41] | **3.27** | [1.85-5.76] | **2.26** | [2.06-2.48] | 0.75 | [0.29-1.95] | **2.21** | [1.33-3.66] | **1.43** | [1.32-1.55] | 1.01 | [0.73-1.41] | 1.09 | [0.69-1.73] |
| All | 35-64 | **1.94** | [1.07-3.5] | **2.91** | [1.98-4.27] | **2.21** | [1.96-2.49] | 0.97 | [0.35-2.69] | 0.94 | [0.41-2.12] | **1.40** | [1.2-1.63] | **2.45** | [1.36-4.39] | 1.15 | [0.64-2.08] |
|  | 65+ | **2.48** | [1.55-3.99] | **3.30** | [2.48-4.39] | **2.32** | [2.14-2.52] | **2.01** | [1.38-2.95] | **1.81** | [1.41-2.33] | **1.49** | [1.4-1.59] | 1.08 | [0.84-1.4] | 1.15 | [0.82-1.6] |
| **10-year follow-up** | |  |  |  |  |  |  |  |  |  |  |  |  |  |  |  |  |
| Males | 35-64 | **1.67** | [1.08-2.58] | **2.14** | [1.55-2.96] | **2.06** | [1.79-2.36] | 1.15 | [0.6-2.2] | 1.01 | [0.54-1.9] | **1.31** | [1.09-1.58] | **2.04** | [1.27-3.27] | 0.66 | [0.36-1.23] |
|  | 65+ | **2.24** | [1.37-3.66] | **2.58** | [1.93-3.44] | **2.17** | [1.94-2.43] | **1.63** | [1.21-2.19] | 1.19 | [0.95-1.49] | **1.35** | [1.24-1.46] | 1.09 | [0.77-1.56] | 1.10 | [0.81-1.51] |
| Females | 35-64 | 1.67 | [0.63-4.46] | **2.67** | [1.44-4.95] | **2.06** | [1.83-2.32] | 1.94 | [0.68-5.59] | 0.55 | [0.21-1.43] | **1.23** | [1.05-1.45] | 0.88 | [0.36-2.16] | 1.10 | [0.43-2.83] |
|  | 65+ | **2.90** | [1.59-5.28] | **3.17** | [1.94-5.16] | **2.26** | [2.11-2.41] | 0.85 | [0.49-1.47] | 1.58 | [0.96-2.59] | **1.37** | [1.29-1.45] | 1.05 | [0.83-1.33] | 1.16 | [0.86-1.57] |
| All | 35-64 | **2.12** | [1.43-3.15] | **2.73** | [2.06-3.63] | **2.11** | [1.93-2.31] | 1.52 | [0.85-2.69] | 1.10 | [0.62-1.95] | **1.30** | [1.16-1.46] | **2.16** | [1.4-3.34] | 0.89 | [0.53-1.49] |
|  | 65+ | **2.88** | [1.96-4.24] | **3.28** | [2.56-4.22] | **2.28** | [2.15-2.42] | **1.84** | [1.4-2.42] | **1.55** | [1.27-1.9] | **1.45** | [1.38-1.51] | 1.14 | [0.94-1.39] | 1.22 | [0.98-1.54] |
| **15-year follow-up** | |  |  |  |  |  |  |  |  |  |  |  |  |  |  |  |  |
| Males | 35-64 | **2.02** | [1.39-2.93] | **2.01** | [1.53-2.63] | **2.07** | [1.85-2.32] | 1.07 | [0.6-1.92] | 0.93 | [0.54-1.59] | **1.27** | [1.08-1.49] | **1.89** | [1.25-2.87] | 1.04 | [0.65-1.67] |
|  | 65+ | **2.01** | [1.32-3.08] | **2.58** | [2.02-3.3] | **2.28** | [2.09-2.5] | **1.58** | [1.24-2.02] | **1.23** | [1.02-1.47] | **1.33** | [1.24-1.43] | 1.15 | [0.85-1.57] | 1.24 | [0.95-1.61] |
| Females | 35-64 | 1.38 | [0.52-3.7] | **2.84** | [1.7-4.74] | **2.07** | [1.88-2.28] | 1.61 | [0.55-4.76] | 0.47 | [0.18-1.22] | **1.19** | [1.03-1.36] | 1.08 | [0.49-2.39] | 1.06 | [0.46-2.43] |
|  | 65+ | **2.69** | [1.49-4.86] | **2.96** | [1.81-4.83] | **2.22** | [2.1-2.35] | 0.89 | [0.54-1.48] | **1.51** | [1.01-2.25] | **1.33** | [1.27-1.4] | 1.09 | [0.91-1.3] | 1.16 | [0.89-1.51] |
| All | 35-64 | **2.41** | [1.7-3.42] | **2.59** | [2.05-3.27] | **2.12** | [1.97-2.28] | 1.36 | [0.8-2.3] | 1.00 | [0.61-1.65] | **1.25** | [1.13-1.38] | **2.06** | [1.4-3.03] | 1.24 | [0.83-1.87] |
|  | 65+ | **2.61** | [1.85-3.68] | **3.17** | [2.53-3.99] | **2.28** | [2.18-2.39] | **1.71** | [1.36-2.15] | **1.53** | [1.3-1.81] | **1.40** | [1.35-1.46] | **1.18** | [1.01-1.38] | **1.26** | [1.04-1.54] |
| Boldface indicates statistical significance (p<0.05) | | | | | | | | | | | | | | | | | |

**Table S8 (Continued)**: Estimated all-cause mortality hazard ratios (HRs) and 95% CIs by years of follow-up, sex, age groups, and tobacco-use status (with never tobacco users as reference group), adjusted by race/ethnicity, education, poverty level, and BMI.

| **Follow-up / Sex** | **Age Group** | **Current Smokers** | | | | | | **Former Smokers** | | | | | | **Never Smokers** | | | |
| --- | --- | --- | --- | --- | --- | --- | --- | --- | --- | --- | --- | --- | --- | --- | --- | --- | --- |
|  |  | **Current SLT users** | | **Former SLT users** | | **Never SLT users** | | **Current SLT users** | | **Former SLT users** | | **Never SLT users** | | **Current SLT users** | | **Former SLT users** | |
|  |  | *(Dual users)* | |  |  | *(Exclusive current smokers)* | | *(Switchers: cigarettes to SLT)* | |  |  | *(Exclusive former smokers)* | | *(Exclusive current SLT users)* | | *(Exclusive former SLT users)* | |
|  |  | **HR** | **95% CI** | **HR** | **95% CI** | **HR** | **95% CI** | **HR** | **95% CI** | **HR** | **95% CI** | **HR** | **95% CI** | **HR** | **95% CI** | **HR** | **95% CI** |
| **20-year follow-up** | |  |  |  |  |  |  |  |  |  |  |  |  |  |  |  |  |
| Males | 35-64 | **1.98** | [1.42-2.77] | **2.18** | [1.73-2.75] | **2.08** | [1.87-2.31] | 1.09 | [0.63-1.88] | 0.87 | [0.53-1.43] | **1.20** | [1.03-1.4] | **1.63** | [1.09-2.43] | 1.02 | [0.68-1.54] |
|  | 65+ | **2.22** | [1.53-3.22] | **2.43** | [1.92-3.07] | **2.28** | [2.1-2.47] | **1.59** | [1.28-1.97] | **1.21** | [1.03-1.43] | **1.33** | [1.25-1.42] | 1.13 | [0.85-1.5] | 1.16 | [0.89-1.5] |
| Females | 35-64 | 1.60 | [0.69-3.71] | **2.84** | [1.77-4.54] | **2.06** | [1.89-2.25] | 1.56 | [0.53-4.6] | 0.54 | [0.23-1.3] | **1.18** | [1.04-1.35] | 1.31 | [0.64-2.67] | 1.32 | [0.65-2.7] |
|  | 65+ | **2.61** | [1.39-4.88] | **3.10** | [1.97-4.87] | **2.20** | [2.09-2.32] | 0.84 | [0.52-1.33] | **1.50** | [1.05-2.15] | **1.31** | [1.26-1.37] | 1.09 | [0.92-1.28] | 1.15 | [0.9-1.48] |
| All | 35-64 | **2.44** | [1.78-3.33] | **2.79** | [2.27-3.42] | **2.11** | [1.98-2.26] | 1.39 | [0.84-2.28] | 0.98 | [0.62-1.54] | **1.21** | [1.1-1.33] | **1.90** | [1.32-2.72] | 1.29 | [0.9-1.84] |
|  | 65+ | **2.75** | [1.99-3.81] | **3.06** | [2.47-3.8] | **2.27** | [2.17-2.36] | **1.68** | [1.36-2.06] | **1.50** | [1.3-1.74] | **1.39** | [1.34-1.44] | **1.17** | [1.01-1.35] | **1.23** | [1.02-1.48] |
| **All follow-up** | |  |  |  |  |  |  |  |  |  |  |  |  |  |  |  |  |
| Males | 35-64 | **1.95** | [1.44-2.63] | **2.18** | [1.76-2.71] | **2.13** | [1.93-2.35] | 1.06 | [0.63-1.78] | 0.89 | [0.56-1.4] | **1.19** | [1.03-1.38] | **1.52** | [1.04-2.23] | 1.02 | [0.7-1.5] |
|  | 65+ | **2.12** | [1.53-2.94] | **2.49** | [2.03-3.06] | **2.18** | [2.02-2.35] | **1.54** | [1.25-1.9] | **1.23** | [1.05-1.43] | **1.32** | [1.24-1.4] | 1.16 | [0.89-1.5] | 1.14 | [0.89-1.47] |
| Females | 35-64 | 1.68 | [0.73-3.87] | **2.76** | [1.75-4.37] | **2.06** | [1.9-2.23] | 1.52 | [0.51-4.49] | 0.57 | [0.25-1.28] | **1.17** | [1.04-1.33] | 1.25 | [0.62-2.53] | 1.36 | [0.69-2.68] |
|  | 65+ | **2.57** | [1.41-4.68] | **3.11** | [2.03-4.76] | **2.13** | [2.03-2.24] | 0.85 | [0.55-1.31] | **1.55** | [1.12-2.14] | **1.30** | [1.25-1.35] | 1.04 | [0.89-1.23] | 1.18 | [0.93-1.49] |
| All | 35-64 | **2.39** | [1.8-3.18] | **2.76** | [2.28-3.34] | **2.14** | [2.01-2.27] | 1.33 | [0.83-2.15] | 1.00 | [0.66-1.52] | **1.20** | [1.1-1.32] | **1.78** | [1.26-2.51] | 1.29 | [0.93-1.8] |
|  | 65+ | **2.66** | [1.99-3.56] | **3.11** | [2.57-3.78] | **2.18** | [2.1-2.27] | **1.63** | [1.33-1.99] | **1.52** | [1.32-1.74] | **1.37** | [1.32-1.41] | **1.15** | [0.99-1.32] | **1.24** | [1.04-1.47] |
| Boldface indicates statistical significance (p<0.05) | | | | | | | | | | | | | | | | | |


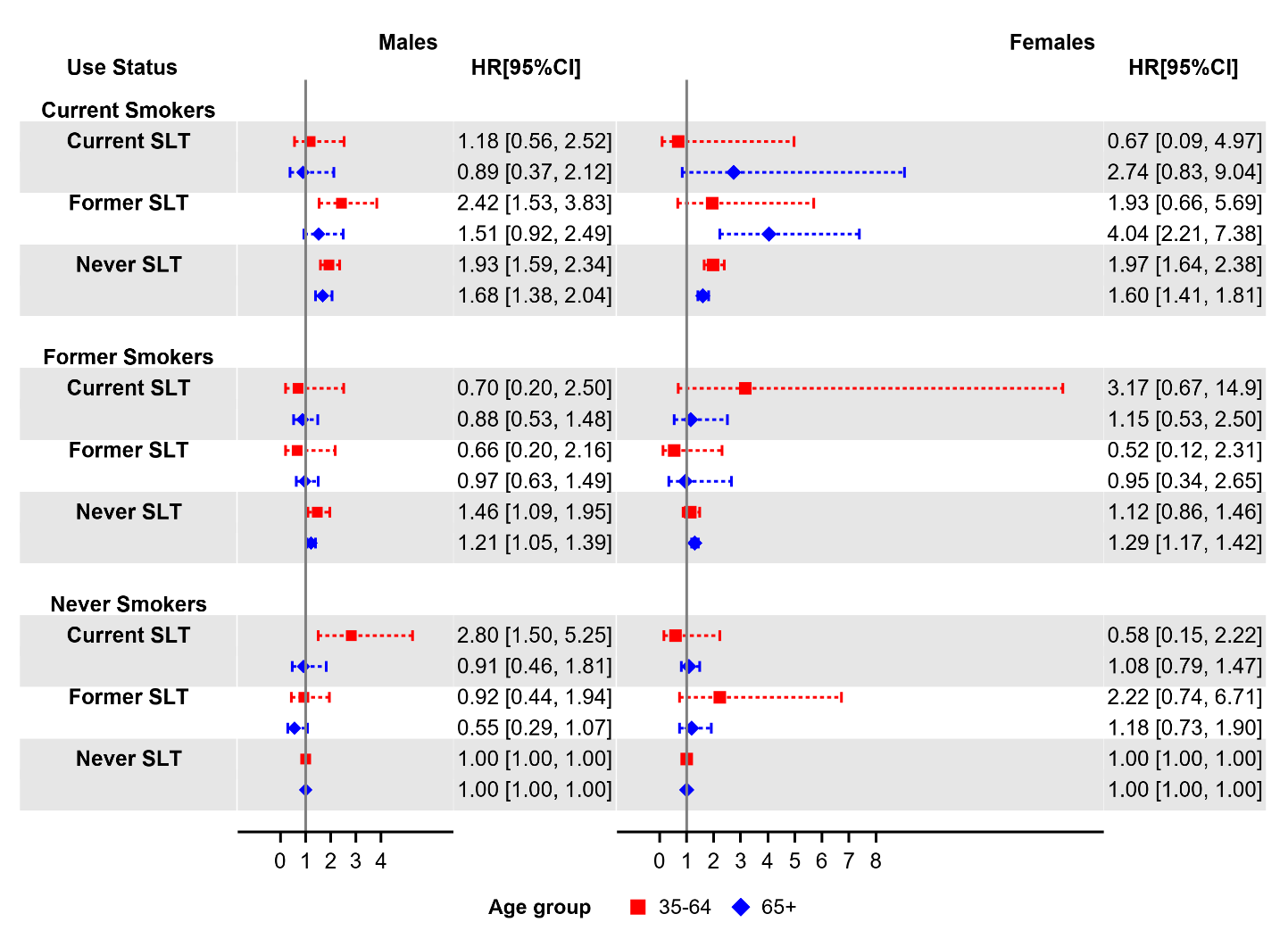


**Figure S1**: Estimated other-cause mortality hazard ratios by sex, age group (35-64 and 65+), and tobacco-use status (with never tobacco users as reference group), adjusted by race/ethnicity, education, poverty level, and BMI. Squares and diamonds indicate the point estimates for age groups 35-64 and 65+, respectively. Horizontal lines represent the length of the 95% CIs.


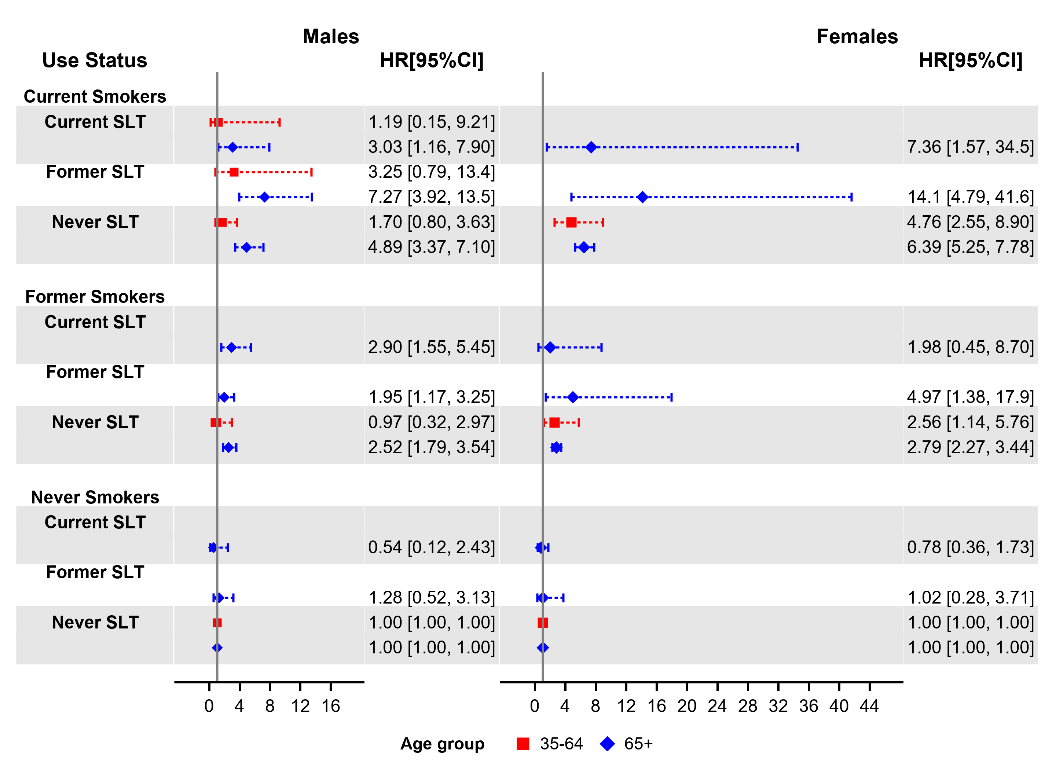


**Figure S2**: Estimated mortality hazard ratios for lung diseases (excluding lung cancer) by sex, age group (35-64 and 65+), and tobacco-use status (with never tobacco users as reference group), adjusted by race/ethnicity, education, poverty level, and BMI. Squares and diamonds indicate the point estimates for age groups 35-64 and 65+, respectively. Horizontal lines represent the length of the 95% CIs.

**References**

1. Levy PS, Lemeshow S. Sampling of Populations: Methods and Applications. 4th ed: Wiley; 2008.
2. Selvin S. Survival Analysis for Epidemiologic and Medical Research: Cambridge University Press; 2008.
