# Supplementary material for: Correction to: Modeling mortality risk effects of cigarettes and smokeless tobacco: results from the national health interview survey linked mortality file data
Source: BMC Public Health. 2022 Jan 4;22:4. doi: 10.1186/s12889-021-12242-1 (PMC8729093; doi:10.1186/s12889-021-12242-1)
Supplement: Supplementary file 1 — Additional file 1: Table S3. Demographic and socioeconomic characteristics (weighted estimates and 95% CIs) for NHIS participants aged 35+ at the time of interview.a [file 12889_2021_12242_MOESM1_ESM.docx]

**Table S3**: Demographic and socioeconomic characteristics (weighted estimates and 95% CIs) for NHIS participants aged 35+ at the time of interview.^a^

| **Variables** | | **Current Smokers** | | | **Former Smokers** | | | **Never Smokers** | | **Never Tobacco Users** | **All** |
| --- | --- | --- | --- | --- | --- | --- | --- | --- | --- | --- | --- |
|  |  | **Current SLT Users** | **Former**  **SLT Users** | **Never SLT Users** | **Current SLT Users** | **Former SLT Users** | **Never**  **SLT Users** | **Current SLT Users** | **Former SLT Users** |  |  |
| **Mean age in years**  (95% CI) | | 48.75  [47.51-49.98] | 48.99  [48.21-49.76] | 51.41  [51.24-51.58] | 56.29  [54.87-57.72] | 57.53  [56.55-58.5] | 60.19  [59.97-60.41] | 53.68  [52.63-54.73] | 50.51  [49.77-51.25] | 54.97  [54.84-55.11] | 55.23  [55.12-55.34] |
| **Sex %** | |  |  |  |  |  |  |  |  |  |  |
|  | Male | 79.11  [73.78-83.60] | 75.25  [71.55-78.61] | 37.56  [36.82-38.30] | 92.08  [89.58-94.01] | 79.68  [76.28-82.71] | 38.23  [37.52-38.95] | 75.81  [72.34-78.98] | 77.1  [73.95-79.98] | 32.53  [32.11-32.95] | 36.25  [35.92-36.57] |
|  | Female | 20.89  [16.40-26.22] | 24.75  [21.39-28.45] | 62.44  [61.70-63.18] | 7.92  [5.99-10.42] | 20.32  [17.29-23.72] | 61.77  [61.05-62.48] | 24.19  [21.02-27.66] | 22.9  [20.02-26.05] | 67.47  [67.05-67.89] | 63.75  [63.43-64.08] |
| **Race/ethnicity %** | |  |  |  |  |  |  |  |  |  |  |
|  | Hispanic | 3.16  [1.91-5.17] | 5.18  [4.00-6.69] | 9.24  [8.81-9.70] | 1.30  [0.47-3.54] | 5.62  [4.11-7.64] | 9.15  [8.71-9.61] | 2.14  [1.28-3.56] | 4.84  [3.79-6.16] | 14.00  [13.58-14.42] | 11.85  [11.51-12.19] |
|  | Non-Hispanic white | 85.38  [81.73-88.4] | 80.95  [78.25-83.38] | 73.51  [72.83-74.18] | 88.25  [84.61-91.12] | 85.54  [82.98-87.77] | 79.28  [78.62-79.92] | 77.35  [73.79-80.55] | 82.23  [79.58-84.61] | 67.47  [66.93-68] | 71.37  [70.91-71.82] |
|  | Non-Hispanic black | 9.80  [7.41-12.85] | 11.33  [9.5-13.46] | 13.5  [12.98-14.04] | 7.80  [5.80-10.42] | 7.58  [6.09-9.4] | 8.39  [7.95-8.84] | 18  [15.29-21.06] | 11.1  [9.13-13.43] | 12.00  [11.63-12.37] | 11.56  [11.23-11.89] |
|  | Non-Hispanic other | 1.66  [0.74-3.68] | 2.54  [1.58-4.05] | 3.74  [3.43-4.08] | 2.65  [1.26-5.49] | 1.27  [0.68-2.33] | 3.18  [2.91-3.48] | 2.52  [1.44-4.35] | 1.83  [1.19-2.8] | 6.54  [6.26-6.83] | 5.23  [5.01-5.45] |
| **Education %** | |  |  |  |  |  |  |  |  |  |  |
|  | Less than high school | 31.85  [26.91-37.23] | 23.05  [20.58-25.71] | 22.31  [21.71-22.92] | 33.82  [29.15-38.82] | 23.66  [20.84-26.73] | 16.71  [16.16-17.27] | 36.76  [33.19-40.48] | 14.18  [12.19-16.44] | 16.17  [15.8-16.55] | 17.70  [17.39-18.03] |
|  | High school diploma | 35.88  [30.9-41.17] | 40.96  [37.34-44.67] | 38.64  [37.93-39.36] | 32.42  [27.64-37.59] | 32.75  [29.32-36.39] | 30.33  [29.69-30.98] | 29.93  [26.47-33.63] | 23.06  [19.94-26.50] | 26.83  [26.44-27.23] | 29.80  [29.47-30.14] |
|  | Some college and higher | 32.28  [26.93-38.14] | 35.75  [32.34-39.32] | 38.50  [37.77-39.23] | 33.50  [28.71-38.66] | 43.48  [39.76-47.27] | 52.46  [51.7-53.22] | 33.04  [29.29-37.02] | 62.44  [58.82-65.92] | 56.36  [55.84-56.88] | 51.91  [51.48-52.35] |
|  | Missing | 0  - | 0.24  [0.08-0.67] | 0.56  [0.46-0.68] | 0.26  [0.05-1.47] | 0.11  [0.02-0.47] | 0.50  [0.40-0.63] | 0.28  [0.10-0.74] | 0.32  [0.11-0.96] | 0.64  [0.57-0.71] | 0.58  [0.53-0.64] |
| **Poverty level %** | |  |  |  |  |  |  |  |  |  |  |
|  | At or above 100% threshold | 76.94  [71.93-81.29] | 76.96  [74.01-79.67] | 73.05  [72.4-73.69] | 78.77  [74.34-82.62] | 83.89  [80.82-86.55] | 79.29  [78.68-79.89] | 72.32  [68.91-75.49] | 82.39  [79.91-84.61] | 77.99  [77.58-78.39] | 77.39  [77.05-77.73] |
|  | Below 100% threshold | 13.03  [9.84-17.07] | 16.11  [13.69-18.86] | 14.08  [13.6-14.58] | 12.13  [9.14-15.92] | 6.39  [4.95-8.21] | 7.01  [6.68-7.37] | 14.63  [12.46-17.1] | 7.38  [5.95-9.13] | 8.62  [8.38-8.87] | 9.39  [9.18-9.61] |
|  | Missing | 10.02  [6.95-14.25] | 6.93  [5.46-8.75] | 12.87  [12.33-13.42] | 9.10  [6.66-12.31] | 9.73  [7.56-12.42] | 13.7  [13.18-14.22] | 13.05  [10.59-15.97] | 10.23  [8.40-12.41] | 13.39  [13.06-13.73] | 13.22  [12.94-13.51] |
| **Body mass index (kg/m^2^) %** | | |  |  |  |  |  |  |  |  |  |
|  | Underweight  (<18.5) | 1.60  [0.66-3.81] | 2.58  [1.70-3.91] | 2.96  [2.74-3.19] | 0.96  [0.34-2.68] | 0.46  [0.21-0.98] | 1.22  [1.09-1.36] | 1.04  [0.61-1.74] | 0.50  [0.25-1.00] | 1.53  [1.43-1.63] | 1.71  [1.64-1.79] |
|  | Normal weight  (18.5-24.9) | 30.58  [25.88-35.72] | 31.06  [27.82-34.49] | 40.94  [40.24-41.64] | 21.39  [17.52-25.84] | 21.56  [18.76-24.64] | 33.1  [32.39-33.81] | 21.61  [18.75-24.76] | 19.66  [17.23-22.34] | 34.84  [34.44-35.24] | 35.19  [34.87-35.5] |
|  | Overweight  (25.0-29.9) | 38.56  [33.47-43.92] | 41.31  [37.83-44.88] | 32.14  [31.47-32.82] | 45.76  [40.59-51.02] | 42.77  [38.86-46.78] | 35.57  [34.87-36.27] | 39.64  [35.9-43.51] | 43.31  [39.87-46.82] | 34.14  [33.75-34.54] | 34.35  [34.04-34.66] |
|  | Obese (30+) | 27.27  [22.55-32.57] | 24  [20.5-27.88] | 21.25  [20.66-21.85] | 30.68  [25.84-35.98] | 33.26  [29.5-37.25] | 26.85  [26.19-27.53] | 35.49  [31.58-39.6] | 35.63  [32.11-39.32] | 25.94  [25.57-26.31] | 25.49  [25.19-25.79] |
|  | Missing | 1.99  [0.89-4.39] | 1.05  [0.51-2.12] | 2.72  [2.49-2.96] | 1.21  [0.56-2.60] | 1.95  [0.98-3.85] | 3.27  [3.00-3.55] | 2.22  [1.51-3.26] | 0.89  [0.45-1.77] | 3.55  [3.39-3.71] | 3.27  [3.14-3.40] |
| ^a^ Excluding participants with missing tobacco-use status, poly-users, and users of other tobacco products including pipe, hookah, e-cigarettes, bidi, and cigars. Survey years: 1987, 1991, 1992, 1994, 1998, 2000, 2005, 2010, and 2012-2014  SLT: smokeless tobacco | | | | | | | | | | | |
